# Insights into malaria susceptibility using genome-wide data on 17,000 individuals from Africa, Asia and Oceania

**DOI:** 10.1038/s41467-019-13480-z

**Published:** 2019-12-16

**Authors:** Malaria Genomic Epidemiology Network, Gavin Band, Quang Si Le, Geraldine M. Clarke, Katja Kivinen, Christina Hubbart, Anna E. Jeffreys, Kate Rowlands, Ellen M. Leffler, Muminatou Jallow, David J. Conway, Fatoumatta Sisay-Joof, Giorgio Sirugo, Umberto d’Alessandro, Ousmane B. Toure, Mahamadou A. Thera, Salimata Konate, Sibiri Sissoko, Valentina D. Mangano, Edith C. Bougouma, Sodiomon B. Sirima, Lucas N. Amenga-Etego, Anita K. Ghansah, Abraham V. O. Hodgson, Michael D. Wilson, Anthony Enimil, Daniel Ansong, Jennifer Evans, Subulade A. Ademola, Tobias O. Apinjoh, Carolyne M. Ndila, Alphaxard Manjurano, Chris Drakeley, Hugh Reyburn, Nguyen Hoan Phu, Nguyen Thi Ngoc Quyen, Cao Quang Thai, Tran Tinh Hien, Yik Ying Teo, Laurens Manning, Moses Laman, Pascal Michon, Harin Karunajeewa, Peter Siba, Steve Allen, Angela Allen, Melanie Bahlo, Timothy M. E. Davis, Victoria Simpson, Jennifer Shelton, Chris C. A. Spencer, George B. J. Busby, Angeliki Kerasidou, Eleanor Drury, Jim Stalker, Alexander Dilthey, Alexander J. Mentzer, Gil McVean, Kalifa A. Bojang, Ogobara Doumbo, David Modiano, Kwadwo A. Koram, Tsiri Agbenyega, Olukemi K. Amodu, Eric Achidi, Thomas N. Williams, Kevin Marsh, Eleanor M. Riley, Malcolm Molyneux, Terrie Taylor, Sarah J. Dunstan, Jeremy Farrar, Ivo Mueller, Kirk A. Rockett, Dominic P. Kwiatkowski

**Affiliations:** 1 Wellcome Centre for Human Genetics, University of Oxford, Roosevelt Drive, Oxford, OX3 7BN UK; 2 Wellcome Sanger Institute, Hinxton, Cambridge, CB10 1SA UK; 3 Li Ka Shing Centre for Health and Information Discovery, Big Data Institute, Old Road Campus, Oxford, OX3 7LF UK; 4 Medical Research Council Unit The Gambia at the London School of Hygiene and Tropical Medicine, Atlantic Boulevard, Fajara, The Gambia; 5 Royal Victoria Teaching Hospital, Independence Drive, Banjul, The Gambia; 6 Faculty of Infectious and Tropical Diseases, London School of Hygiene and Tropical Medicine, Keppel Street, London, UK; 7 Malaria Research and Training Centre, Faculty of Medicine, University of Bamako, Bamako, Mali; 8 University of Rome La Sapienza, Roma, Italy; 9 Centre National de Recherche et de Formation sur le Paludisme (CNRFP), Ouagadougou, Burkina Faso; 10 Navrongo Health Research Centre, Navrongo, Ghana; 11 Noguchi Memorial Institute for Medical Research, Accra, Ghana; 12 Komfo Anokye Teaching Hospital, Kumasi, Ghana; 13 Kwame Nkrumah University of Science and Technology, Kumasi, Ghana; 14 Department of Molecular Medicine, Bernhard Nocht Institute for Tropical Medicine, Postfach 30 41 2, D-20324 Hamburg, Germany; 15 Kumasi Centre for Collaborative Research, Kumasi, Ghana; 16 Institute of Child Health, College of Medicine, University of Ibadan, Ibadan, Nigeria; 17 Department of Biochemistry and Molecular Biology, University of Buea, Buea, South West Region Cameroon; 18 KEMRI-Wellcome Trust Research Programme, PO Box 230, Kilifi, Kenya; 19 Joint Malaria Programme, Kilimanjaro Christian Medical Centre, PO box 2228, Moshi, Tanzania; 20 Mwanza Research Centre, National Institute for Medical Research, Mwanza, Tanzania; 21 Oxford University Clinical Research Unit, 764 Vo Van Kiet, District 5, Ho Chi Minh City, Vietnam; 22 Hospital for Tropical Diseases, 764 Vo Van Kiet, District 5, Ho Chi Minh City, Vietnam; 23 Saw Swee Hock School of Public Health, National University of Singapore, Tahir Foundation Building, 12 Science Drive 2, #10-01, Singapore, 117549 Singapore; 24 Papua New Guinea Institute of Medical Research, PO Box 60, Garoka, EHP441 Papua New Guinea Guinea; 25 University of Western Australia, Perth, Western Australia Australia; 26 Faculty of Medicine and Health Sciences, Divine Word University, Madang, Papua New Guinea; 27 The Walter and Eliza Hall Institute of Medical Research, Melbourne, Victoria Australia; 28 Liverpool School of Tropical Medicine, Pembroke Place, Liverpool, L3 5QA UK; 29 Weatherall Institute of Molecular Medicine, Oxford University, Oxford, UK; 30 The Ethox Centre, Nuffield Department of Population Health, University of Oxford, Old Road Campus, Oxford, OX3 7LF UK; 31 Wellcome Centre for Ethics and Humanities, Nuffield Department of Population Health, Big Data Institute, Li Ka Shing Centre for Health and Information Discovery, Old Road Campus, Oxford, OX3 7LF UK; 32 Institute of Medical Microbiology, University Hospital of Dusseldorf, Dusseldorf, North Rhine-Westphalia Germany; 33 Genome Informatics Section, Computational and Statistical Genomics Branch, National Human Genome Research Institute, Bethesda, Maryland USA; 34 Department of Medical Laboratory Sciences, University of Buea, Buea, South West Region Cameroon; 35 Faculty of Medicine, Department of Medicine, Imperial College, Exhibition Road, London, SW7 2AZ UK; 36 Nuffield Department of Medicine, NDM Research Building, Roosevelt Drive, Headington, Oxford, OX3 7FZ UK; 37 The Roslin Institute, The University of Edinburgh, Easter Bush Campus, Midlothian, EH25 9RG UK; 38 Malawi-Liverpool-Wellcome Trust Clinical Research Programme, College of Medicine, University of Malawi, PO Box 30096, Blantyre, Malawi; 39 Blantyre Malaria Project, College of Medicine, University of Malawi, Blantyre, Malawi; 40 College of Osteopathic Medicine, Michigan State University, East Lansing, Michigan 48824 USA; 41 Peter Doherty Institute for Infection and Immunity, The University of Melbourne, Melbourne, Victoria Australia; 42 Centre for Tropical Medicine, Nuffield Department of Clinical Medicine, Oxford University, Oxford, OX3 7LJ UK; 43 Barcelona Centre for International Health Research (CRESIB), Barcelona, Spain

**Keywords:** Genome-wide association studies, Medical genomics, Immunogenetics, Malaria

## Abstract

The human genetic factors that affect resistance to infectious disease are poorly understood. Here we report a genome-wide association study in 17,000 severe malaria cases and population controls from 11 countries, informed by sequencing of family trios and by direct typing of candidate loci in an additional 15,000 samples. We identify five replicable associations with genome-wide levels of evidence including a newly implicated variant on chromosome 6. Jointly, these variants account for around one-tenth of the heritability of severe malaria, which we estimate as ~23% using genome-wide genotypes. We interrogate available functional data and discover an erythroid-specific transcription start site underlying the known association in *ATP2B4*, but are unable to identify a likely causal mechanism at the chromosome 6 locus.  Previously reported HLA associations do not replicate in these samples. This large dataset will provide a foundation for further research on the genetic determinants of malaria resistance in diverse populations.

## Abstract (web summary)

Four genome-wide associated loci are currently known for malaria susceptibility. Here, the authors expand on earlier work by combining data from 11 malaria-endemic countries and additional population sequencing informing an African-enriched imputation reference panel, with findings including a previously unreported association on chromosome 6.

## Introduction

Genome-wide association studies (GWASs) have been very successful in identifying common genetic variants underlying chronic non-communicable diseases, but have proved to be more difficult for acute infectious diseases that represent a substantial portion of the global disease burden and are most prevalent in tropical regions. This is partly due to the practical difficulties of establishing large sample collections and reliable phenotypic datasets in resource-constrained settings, but also theoretical and methodological challenges associated with the study of pathogenic diseases in populations with high levels of genetic diversity and population structure^1–3^. The Malaria Genomic Epidemiology Network (MalariaGEN) was established in 2005 to overcome these obstacles with standardized protocols, common phenotypic definitions, agreed policies for equitable data sharing and local capacity building for genetic data analysis, enabling large collaborative studies across different countries where malaria is endemic^4^.

Here we extend previous work by using data collected from 11 countries to perform a comprehensive GWAS of human resistance to severe malaria (SM). We incorporate DNA from 17,056 SM cases and population controls genotyped and statistically phased at over 1.5 million single-nucleotide polymorphisms (SNPs) across the human genome, and impute from a modified version of the 1000 Genomes genetic variation reference panel enriched with 773 additional African genome sequences, plus locus-specific panels in the glycophorin and human leukocyte antigen (HLA) regions. We analyse association in a Bayesian meta-analysis framework, allowing for differences between populations and subphenotypes, and identify five loci with genome-wide levels of evidence which are further supported in a set of replication samples and jointly explain ~10% of the estimated heritability. Among these, a newly implicated locus on chromosome 6 appears most strongly associated with cerebral malaria (CM), but lies over 700 kb from the nearest protein-coding gene. We analyse available functional data, including RNA-sequencing (RNA-seq) from erythroid lineage cells, but are unable to identify a putative function for this locus. However, this analysis does provide insights into the potential mechanism of protection at the previously reported *ATP2B4* locus, via disruption of GATA1-mediated expression of an erythroid-specific transcript. Across all loci, empirical models of allele frequencies suggest there has been a systematic positive selection of malaria-protective alleles and we conclude by noting that coevolutionary selection pressure may also affect *Plasmodium falciparum* populations, raising the need to incorporate parasite genetic variation into future studies.

## Results

### Human genomes reflect diversity in malaria-endemic regions

We generated genome-wide SNP typing on samples from our previously described case–control study, which includes records for over 16,000 individuals admitted to a hospital with severe symptoms of *P. falciparum* malaria, and 22,000 population controls^5^. In brief, individuals were sampled from 12 study sites, including 10 in sub-Saharan Africa and the others in Vietnam and Papua New Guinea (PNG; Supplementary Table 1). SM was ascertained according to the World Health Organization (WHO) criteria^6^ and represents a heterogeneous phenotype including diagnoses of CM, SM anaemia (SMA) and other malaria-related symptoms (here referred to as other SM). For our GWAS discovery phase, a subset of samples, preferentially chosen for phenotype severity and DNA quality, were genotyped on the Illumina Omni 2.5 M platform. We jointly processed these samples to produce a single set of estimated haplotypes for 17,960 individuals at the set of over 1.5 M SNPs genome-wide which passed our quality control process (Methods). Subsets of these data from The Gambia, Malawi and Kenya^7^, from Tanzania^8^ and from selected control samples^9^ have been reported previously (Supplementary Table 2). In total, this dataset includes 6888 individuals from Mali, Burkina Faso, Ghana, Nigeria, Cameroon, Tanzania, Vietnam and PNG, which have not previously been included in meta-analysis, and reflects the haplotype diversity of a substantial portion of the malaria-endemic world.

### Improved genotype imputation through population sequencing

**Fig. 1:**
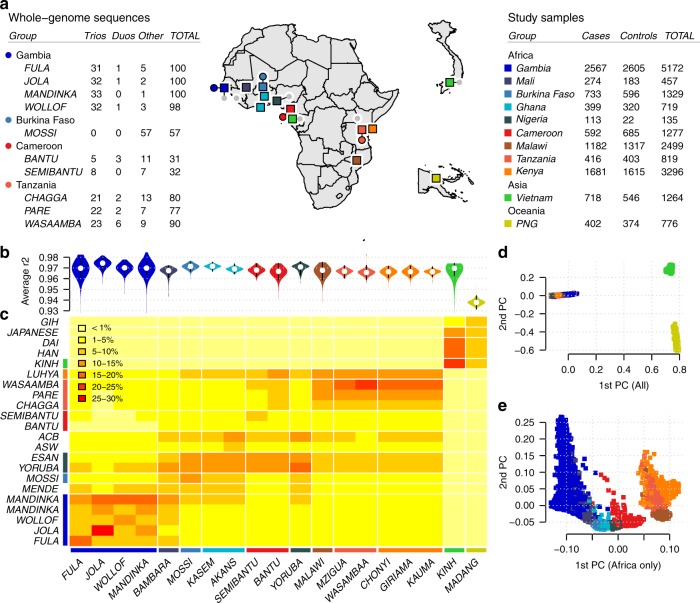
Overview of datasets and imputation performance. **a** Counts of whole-genome sequenced samples (reference panel samples, left table), samples typed on the Omni 2.5 M platform (study samples, right table) and geographic locations of sampling (map). Counts reflect numbers of samples following our quality control process. Sequenced samples were collected in family trios, except in Burkina Faso, as shown. Colours shown in tables and map denote country of origin of reference panel (circles) and study samples (squares), with small grey circles indicating 1000 Genomes Project populations. **b** Imputation performance, measured as the mean squared correlation between directly typed and re-imputed variants for each sample. **c** Distribution of the most similar haplotypes. For each GWAS sample, the average number of 1 Mb chunks such that the most similar haplotype lies in the given reference panel population (y axis) is shown. Values are averaged over samples within each GWAS population (x axis). **d**, **e** Principal components (PCs) computed across 17,120 study samples identified without close relationships, or the subset of 15,152 samples of African ancestry.

The ethnically diverse nature of our study provides challenges for genomic inference, including for our ability to impute genotypes at potentially relevant untyped loci^10^. To address this, we sequenced the genomes of 773 individuals from ten ethnic groups in east and west Africa (specifically from the Gambia, Burkina Faso, Cameroon and Tanzania), including 207 family trios (Fig. 1). We combined genotypes at SNPs in these data with Phase 3 of the 1000 Genomes Project to form an imputation reference panel, which covers the most common genetic variation^11^ and in which two-fifths of the donor families are of African ancestry (1203 of 3046 individuals). In principle, the additional representation of African DNA in this panel should lead to improvements in imputation accuracy for African study populations and we found that this was indeed the case, with the use of our panel leading to a large increase in accuracy relative to panels used in our previous GWAS^7,10,12^ and a more modest improvement relative to using the 1000 Genomes Phase 3 panel alone (Supplementary Fig. 1). Imputation of Vietnamese individuals and those from PNG, which is substantially diverged from any reference panel population (Fig. 1d), were less affected by the inclusion of these additional haplotypes.

We specifically examined imputation of malaria-protective alleles in the *HBB* gene, which have previously been found difficult to impute^10,12^. The SNP encoding the sickle cell mutation (rs334, chr11:5248232) was imputed with *r* > 0.9 in all African populations, as compared with genotypes obtained through direct typing^5^. We did note that some potentially relevant loci still appear not to be accessible through these data, including the common deletion of *HBA1-HBA2* that causes alpha-thalassaemia (Supplementary Note 1) and the region around the gene encoding the invasion receptor basigin^13^ (Supplementary Note 2). Thus, although overall accuracy is high across the genome, additional work will be needed to access a smaller set of complex but potentially relevant regions.

### Association testing implicates a new locus on chromosome 6

**Fig. 2:**
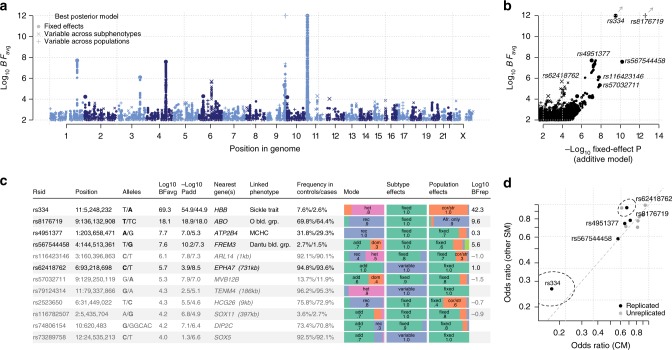
Evidence for association with severe malaria. **a** Association evidence (log10 BF_avg_, y axis; clamped to a maximum of 12) at typed and imputed SNPs and indels genome-wide (x axis). BF_avg_ reflects evidence under a range of models summarized using prior weights specified in Methods. Shapes denote whether the model with the highest posterior weight is for effects fixed across populations and subphenotypes (case–control effect, circles), or suggests variation in effect between populations (crosses) or between subphenotypes (plusses). **b** Comparison of model-averaged Bayes factor (log10 BF_avg_, y axis) and the evidence under an additive model of association with overall SM (−log10 P_add_, x axis). For visualization purposes, we have removed variants in the region of rs334 (HbS, chromosome 11) and rs567544458 (glycophorin region, chromosome 4) except the lead variant. Shapes are as in **a**. The values for rs334 and rs8176719 lie outside the plot as indicated by arrows; to visualize these we have projected them onto the plot boundary. **c** Twelve regions of the genome with BF_avg_ > 10,000. Columns reflect the ID, genomic position, reference, and alternative allele with estimated protective allele indicated in bold, log10 BF_avg_, −log10 *P*-value for an additive model of association with SM or with SM subtypes, nearest gene and distance to the nearest gene for intergenic variants, known linked phenotypes and combined protective allele frequency across African control and case samples. Bar plots summarize our inference about the mode of effect of the protective allele and the distribution of effects between SM subtypes and between populations. The last column reflects the evidence for association observed in replication samples (log10 BF_replication_), assessed using the effect-size distribution learnt from discovery samples, based on direct typing of tag SNPs as detailed in Supplementary Data 1. Rows are in bold if they showed positive replication evidence (BF_replication_ > 1). **d** Comparison of estimated effect sizes for the protective allele on CM (y axis) and on unspecified SM cases (x axis) for the 12 variants in **c**. The 95% confidence region for rs334 and rs62418762 (dashed ellipses) are shown. Source data are provided as a Source Data file.

We used the imputed genotypes to test for association with SM and with SM subtypes, at over 15 million SNPs and indels genome-wide, using a subset of 17,056 SM cases and controls identified without close relationships (Methods). Specifically, association tests were conducted within each population using logistic and multinomial logistic regression (implemented in SNPTEST; Methods), including principal components (PCs) to control for population structure, and we computed a fixed-effect meta-analysis summary of association across populations (Supplementary Fig. 3). In view of the complexities observed at known association signals^10,12,14^, we also extended our previously described meta-analysis method^5,7^ to the larger set of populations, phenotypes and variants considered here. This analysis produces an overall measure of evidence (the model-averaged Bayes factor, BF_avg_; Fig. 2a) that is sensitive to more complex patterns of genetic effect than are detected by frequentist fixed-effect analysis (Fig. 2b). The BF_avg_ is computed under specific prior weights that are detailed in Methods. In particular, BF_avg_ captures effects that have non-additive mode of inheritance, as well as effects that are restricted to particular subphenotypes or that vary between populations.

**Fig. 3:**
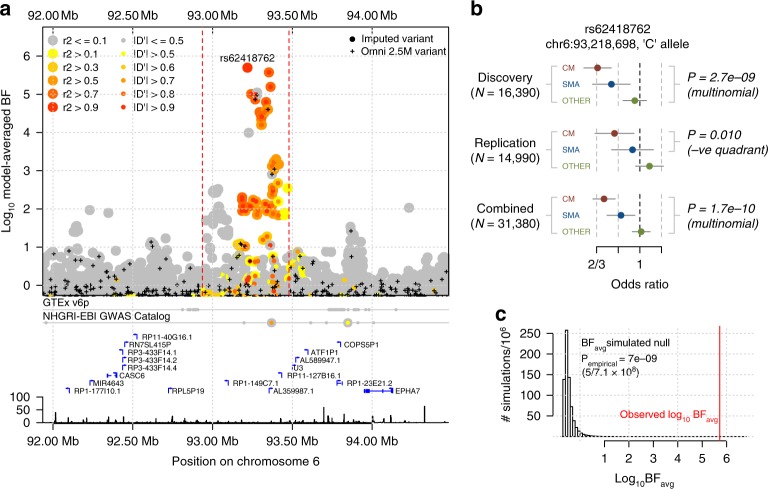
Evidence for association at rs62418762. **a** Regional hitplot showing evidence for association (log10 BF_avg_, y axis) across a 2.5 Mb region surrounding rs62418762 (x axis). Points are coloured by LD with rs62418762, estimated using African reference panel haplotypes. Directly typed SNPs included in the phased dataset are denoted by black plusses. Below, the locations of significant tissue-specific eQTLs, previously identified association signals, regional genes, pseudogenes and noncoding RNAs, and the Hapmap-combined recombination rate map are annotated. **b** Detail of discovery and replication evidence for association at rs62418762 under an additive model. Points and lines represent the estimated odds ratio of the ‘C’ allele on severe malaria subtypes. Estimates are obtained using multinomial logistic regression in each population and combined across populations using fixed-effect meta-analysis. Top: effect sizes estimated from imputed genotypes in discovery samples. A Wald test *P*-value against the null that all three effect sizes are zero is shown. Middle: effect sizes estimated from direct typing of rs62418762 in replication samples. *P*-value reflects the alternative hypothesis that CM and SMA effects are nonzero and in the direction observed in discovery and is computed by simulation. Bottom: meta-analysis of discovery and replication results. **c** Empirical null distribution of the discovery BF_avg_, computed using simulations conditional on the observed frequencies of rs62418762. The red line indicates the observed BF_avg_ and an empirical *P*-value from the simulated distribution is shown. Source data are provided as a Source Data file.

Several regions of the genome showed evidence for association in this analysis, including 7 regions with BF_avg_ > 1 × 10^5^, 12 with BF_avg_ > 1 × 10^4^ (Fig. 2c), and 97 with BF_avg_ > 1000 (Supplementary Data 1). Among these, our data convincingly replicate previously confirmed associations at *HBB*, *ABO*, *ATP2B4*, and in the glycophorin region on chromosome 4, while an additional previously reported variant in *ARL14* also shows stronger evidence in this larger sample^7^. Both a direct interpretation of the Bayes factor as well as false discovery rate (FDR) methods suggest a relatively small number of our top signals may represent real associations (e.g., roughly 5–9 associations among the top 97 given plausible prior odds^15^ of 10^−6^ to 10^−5^; 5 regions meeting FDR < 5% based on the multinomial test *P*_add_). Compatible with this, direct genotyping of 15,548 cases and controls not included in our GWAS discovery phase revealed evidence of replication (determined as BF_replication_ > 1; Methods) for one newly identified locus (rs62418762; BF_replication_ = 9.8; replication *P*_additive_ = 0.01 for an additive protective effect of the ‘C’ allele on CM and SMA; Fig. 3b and Supplementary Fig. 5) from among 20 that we attempted to replicate, in addition to previously reported regions (Supplementary Data 1).

rs62418762 has the second strongest evidence of all SNPs in our discovery phase and the strongest evidence overall (BF_avg_ = 5.0 × 10^5^; multinomial test *P*_add_ = 2.8 × 10^−9^; combined BF = 4.9 × 10^6^; combined *P*_add_ *=* 1.7 × 10^−10^; Fig. 2c and Fig. 3) outside regions of previously confirmed associations. Our estimates suggest the rs62418762 ‘C’ allele is associated with decreased risk of CM (OR_CM_ *=* 0.79, 95% confidence interval (CI) = 0.66–0.95; estimated using only replication samples; Fig. 3b), but less strongly with other malaria subtypes (OR_SMA_ = 0.93, 95% CI = 0.77–1.13; OR_OTHER_ = 1.10, 95% CI = 0.96–1.25). rs62418762 lies in a region of chromosome 6 between *MAP3K7* and the nearest gene *EPHA7*, which encodes Ephrin type-A receptor 7, a regulator of neurodevelopment and neural cell adhesion^16^. Other type-A ephrin receptors have been implicated in liver-stage malaria^17^. However, rs62418762 lies over 700 kb distant from *EPHA7* and it is not immediately clear whether any functional link between them exists. A number of non-protein-coding transcripts lie nearby (e.g., the pseudogene *ATF1P1*), as do reported signals of association with neurodegenerative disorders^18,19^, and these may provide clues as to an underlying mechanism. However, we caution that no functional effect of rs62418762 is apparent at present and additional replication may therefore be warranted.

### Malaria-associated loci show substantial heterogeneity

**Fig. 4:**
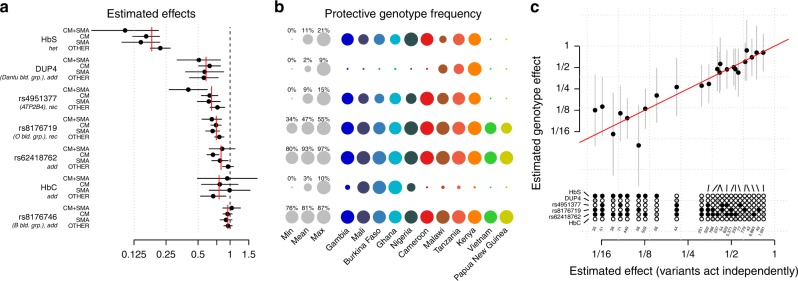
A joint model for natural genetic resistance to malaria. **a** Effect sizes for severe malaria subtypes are estimated in a joint model, which includes the five replicating associated variants and two additional variants (rs33930165, which encodes haemoglobin C, and rs8176746, which reflects the A/B blood group) in associated regions. The model was fit across all 11 populations, assuming the effect on each phenotype is fixed across populations, and including a population indicator and five principal components in each population as covariates. Each variant is encoded according to the mode of inheritance of the protective allele inferred from discovery analysis. Red lines indicate the overall effect across severe malaria subtypes, computed as an inverse variance-weighted mean of the per-phenotype estimates. Only cases with positive measured falciparum parasitaemia were included in model fit. **b** The frequency of the protective allele (for effects inferred as additive) or protective genotype (for non-additive effects) of each variant in each population. Grey circles depict the minimum, mean and maximum observed frequencies across populations. Coloured circles reflect the per-population frequencies. Frequency estimates are computed using control samples only. **c** Comparison of effect-size estimates against severe malaria for combinations of genotypes (stacked circles) carried by at least 25 study individuals, across the top six variants in **a**. Black filled and open circles denote the protective and risk dosage at the corresponding variant, respectively; grey circles denote heterozygote genotype for variants with inferred additive effect. Effect-size estimates are computed using the model as in **a** assuming independent effects (x axis), or jointly allowing each genotype its own effect (y axis). Source data are provided as a Source Data file.

We examined the nature of association at replicated loci in more detail (Figs. 2 and 4) and observed considerable heterogeneity in mode of effect and across populations and subphenotypes. The sickle haemoglobin allele (HbS, encoded by rs334) is present in all African populations studied, but varies considerably in estimated effects, with greater than fourfold difference between the strongest estimate (OR = 0.10 in the Gambia) and the weakest (OR = 0.47 in Cameroon). This does not seem to be explained by the presence of the competing HbC mutation in West African populations (Fig. 4), which is at low frequency in Cameroon. The O blood group-encoding mutation rs8176719 is common in all populations but, in our analysis, appears to have the greatest protective effect in Africa, with an opposite direction of effect observed in PNG, an unexpected feature that is also observed in replication samples (rs8176719, Supplementary Fig. 5), and could point to hitherto unexplained subtleties in the mechanism of protection of this allele^20,21^. The structural variant DUP4, which encodes the Dantu NE blood group phenotype^14^ and is in linkage disequilibrium (LD) with rs567544458 in our genome-wide imputation (Fig. 2c), is essentially absent from study populations outside east Africa (*f* < 0.1%, except in Malawi, Tanzania and Kenya). Our data also demonstrate differences in effect size between malaria subphenotypes, with all of the replicating variants displaying smaller effects in nonspecific cases of SM compared with cerebral or severely anaemic cases (Figs. 2d and 4a). This likely reflects the mixed phenotypic composition of this set of samples and contributes to the trend where replication effect sizes are smaller than those in discovery (Supplementary Figs. 5 and 6). Inclusion of the associated variants in a joint model suggests they act largely independently in determining malaria risk (Fig. 4c).

A longer list of regions showing evidence for heterogeneous effects between populations can be found in Supplementary Data 2. We note that assessing population variation in subphenotype-specific effects is challenging due to the relatively small sample sizes involved, as exemplified by the wide confidence intervals on per-population estimates for rs62418762 (Supplementary Fig. 5), which accordingly does not display strong evidence for between-population heterogeneity in our data (Methods). To avoid possible effects of overfitting at SNPs across the genome, we did not include models of between-population heterogeneity for subphenotypes in our computation of BF_avg_.

### Malaria risk is influenced by a polygenic component

We used published methods to estimate the heritability of SM captured by genome-wide genotypes (e.g., heritability explained by SNPs on the genotyping array (*h*^2^) = 0.23, 95% CI = 0.16–0.30; computed across African populations using PCGC^22^ assuming a 1% population prevalence of SM^23^; Supplementary Fig. 4 and Supplementary Data 3). The *HBB*, *ABO*, glycophorin and *ATP2B4* loci appear to contribute around 11% of this total (i.e., around 2.5% of liability scale phenotype variation) and we noted a trend for the remaining heritability to concentrate near protein-coding genes and at lower-frequency variants (Supplementary Fig. 4d). A number of caveats apply to these estimates, which assume a particular relationship between effect size, allele frequency and the degree of LD surrounding causal variants, all of which may be distorted by effects of natural selection^24,25^, and may further be affected by unmeasured environmental confounders. Nevertheless, our results are comparable to previously reported estimates from family-based studies in east Africa^26^ and suggest that further susceptibility loci will be discoverable with additional data. To assist researchers to integrate our data with other sources of information, including future genetic association studies and functional experiments, the raw and imputed data as well as a full set of results from our analysis are being made available (see Data availability).

### An erythroid-specific transcript underlies *ATP2B4* protection

We reasoned that functional annotations might provide clues to further putatively causal variants among our list of most associated regions. To assess this, we annotated all imputed variants with information indicative of functional importance, including location and predicted function within genes^27^, chromatin state^28–30^ and transcriptional activity^29,31,32^ across cell types, status as an expression quantitative trait loci (eQTL)^32–34^ and association evidence from other traits^35–38^. This analysis revealed a number of potentially functional variants with evidence of association (Supplementary Data 4).

**Fig. 5:**
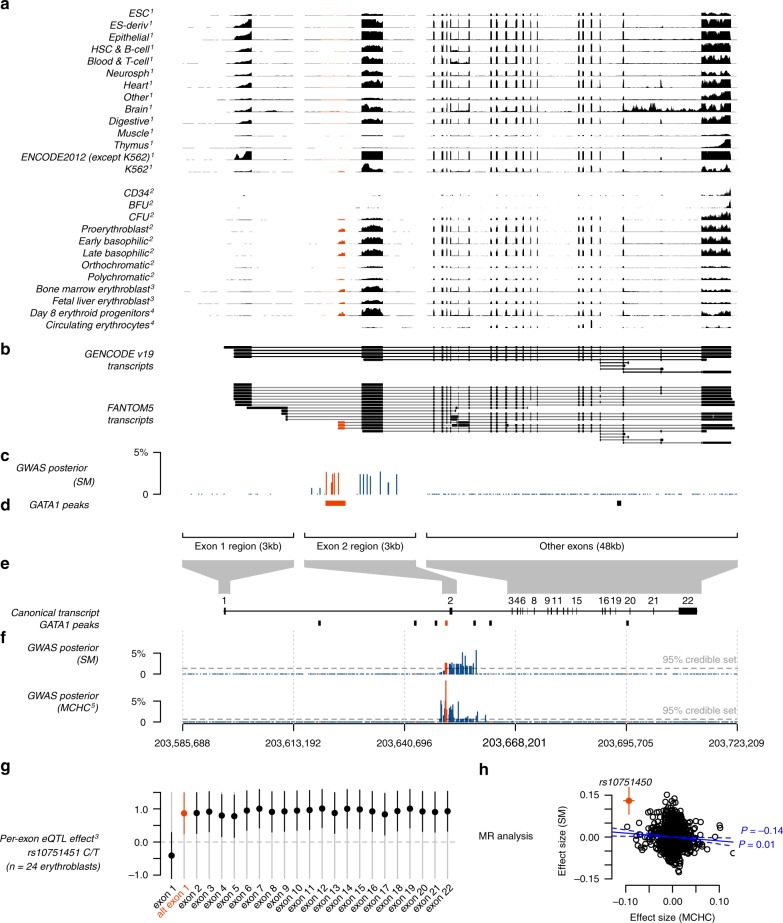
The *ATP2B4* association is driven by an erythrocyte-specific transcription start site. **a** Normalized RNA-seq coverage for (1) 56 cell types from Roadmap Epigenomics^29^ and ENCODE, (2) human CD34^+^ hematopoietic stem and progenitor cells, and experimentally differentiated erythroid cells from three biological replicates^31^, (3) ex-vivo differentiated adult and fetal human erythroblasts from 24 individuals^32^ and (4) experimentally differentiated erythroid progenitor cells and circulating erythrocytes^45^. Coverage is shown across expanded regions of *ATP2B4* exon 1, exon 2 including the putative alternative first exon (located at 203,651,123–203,651,366) and the remaining exons. Throughout, red features are those lying within 500 bp upstream to 50 bp downstream of the alternative first exon. For Roadmap and ENCODE data, the plot reflects normalized coverage maximized across cell types in each tissue group. For other cells, coverage is summed over samples and normalized by the mean across *ATP2B4* exons. **b** *ATP2B4* transcripts from the GENCODE^41^ and FANTOM5^44^ transcript models. **c** Posterior evidence for association with SM assuming a single causal variant. **d** Position of GATA1-binding peaks^28^. **e** Location and size of the expanded regions shown against the full-length transcript, with GATA1-binding peaks shown. **f** Posterior evidence for association with SM as in **c** and with mean corpuscular haemoglobin concentration (MCHC)^36^, assuming a single causal variant for each trait separately. **g** Estimated effect of rs10751451 on each exon of *ATP2B4*, computed by linear regression against FPKM residuals after correcting for cell development stage^32^, with 95% confidence intervals shown. For comparable visualization across exons, FPKM is further normalized by the mean across samples at each exon. **h** Mendelian randomization analysis of SM and MCHC at 2130 ‘sentinel’ SNPs previously identified as associated with haematopoetic traits^36^ with association results in our study. Points reflect the posterior effect-size estimates on SM (y axis) and MCHC (x axis), conditional on the fitted bivariate Gaussian model of effect sizes. Variants are assumed to act independently. Blue solid and dotted lines and text show the maximum likelihood estimate of the effect of MCHC on SM (*ρ*), its 95% confidence interval, and likelihood ratio test *P*-value against the null that *ρ* = 0.

We uncovered compelling evidence that the association in *ATP2B4*, which encodes the plasma membrane calcium pump PMCA4, is driven by an alternative transcription start site (TSS) that is specific to erythroid cells (Fig. 5). Specifically, we found that associated SNPs overlap a binding site for the GATA1 transcription factor, an important regulator of expression in erythroid cells^28,30,39^, in the first intron of *ATP2B4*. The derived allele at one of these SNPs (rs10751451) disrupts a GATA motif^32^ and the same SNPs have previously been shown to associate with *ATP2B4* expression levels in whole blood^34^, in experimentally differentiated erythrocyte precursors^32^ and with PMCA4 levels in circulating red blood cells (RBCs)^40^. We noted that the GATA1 site lies just upstream of an exon that is not listed in the GENCODE^41^, RefSeq^42^ or ENSEMBL^43^ transcript models, but that can be found in FANTOM5^44^ (Fig. 5b). Inspection of RNA-seq data revealed that this forms the first exon of the main *ATP2B4* transcript expressed by erythroid cells^30,32,45^, as well as by the K562 cell line, but appears not to be expressed in other cell types^28,29,33^ (Fig. 5a). Moreover, the derived allele, which is associated with malaria protection, is correlated with decreased expression of this alternative first exon (*P* = 0.01, using data from 24 fetal and adult erythroblasts^32^; Fig. 5g) and all subsequent exons (*P* < 0.03) of *ATP2B4*, but not with the annotated first exon (*P* = 0.25). These results indicate that the malaria-associated SNPs affect *ATP2B4* expression in a erythroid-specific manner, by affecting the promoter of a TSS that is only active in these cells.

The situation outlined above for *ATP2B4* is reminiscent of the well-known mutation at the Duffy blood group locus (*DARC/ACKR1*), which protects against *Plasmodium vivax* malaria by preventing erythrocytic expression of the Duffy antigen receptor through disruption of a GATA1-binding site^46,47^. However, the mechanism by which *ATP2B4* affects parasite processes remains unknown. The malaria-protective allele at rs10751451 is known to associate with RBC indices (notably with increased mean corpuscular haemoglobin concentration (MCHC)^32,36,48^. We found some evidence that genetic predisposition to high MCHC levels may itself be associated with decreased malaria susceptibility (negative observed correlation between MCHC and SM effect size at sentinel SNPs previously identified as associated with haematological indices in European individuals^36^, *ρ* = − 0.14, *P* = 0.01; in a Mendelian randomization (MR) framework; Fig. 5h). However, the protective effect at *ATP2B4* appears substantially stronger than this trend and suggests a genuinely pleiotropic effect whose mechanism against malaria is yet to be determined.

Our analysis of functional annotations revealed a number of additional variants with some evidence of function. These include variants in the HLA region, discussed further below, and a variant upstream of *VAC14*, which has been implicated in Typhoid fever^49^ and *Salmonella typhi* invasion^35^ detailed further in Supplementary Note 4.

### Classical HLA alleles appear unassociated with malaria risk

**Fig. 6:**
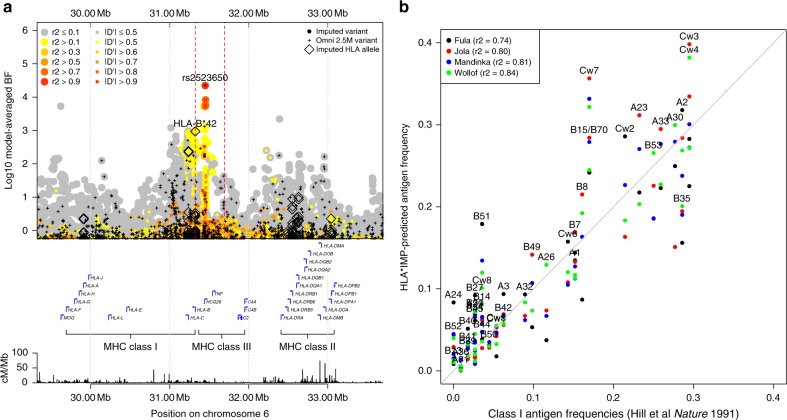
Evidence for association across the HLA. **a** Evidence for association at genotyped SNPs (black plusses), imputed SNPs and INDELs (circles), and imputed classical HLA alleles (black diamonds) across the HLA region. Points are coloured by LD with rs2523650 as estimated in the African reference panel populations. Selected regional genes and the Hapmap-combined recombination map are shown below. **b** Comparison of HLA Class I antigen frequencies in distinct sample sets from The Gambia. X axis: antigen frequencies obtained by serotyping of 112 healthy adults^51^; y axis: inferred antigen frequencies based on imputation of 2-digit alleles in the four major ethnic groups (colours) in our Gambian dataset. B*15 alleles encode a number of antigens^100^ including B70 and we combine results for B15 and B70 here.

Motivated by the modest evidence for association observed in the HLA (Supplementary Data 1), we used a published method^50^ to impute classical HLA alleles and tested for association with each allele as described above for genome-wide variants (Fig. 6a). The strongest evidence for association with an HLA antigen was observed at HLA-B*42 (BF_avg_ = 834; synonymous with HLA-B*42:01 in this panel). By contrast, an order of magnitude stronger evidence was observed at regional SNPs, including rs2523650 (BF_avg_ = 1.9 × 10^4^; recessive model OR = 0.85 for the non-reference ‘C’ allele; 95% CI = 0.79–0.90; *P*_add_ = 4.7 × 10^−7^; Supplementary Fig. 6), which has previously been associated with blood cell traits and expression of regional genes (Supplementary Data 4), but this signal did not replicate in additional samples (BF_replication_ = 0.19). Most notably, the HLA-B*53 allele, which has previously been reported as associated with strong protection against SM in the Gambia^51^, showed no evidence for association across populations (BF_avg_ = 0.17; OR = 1.0, 95% CI = 0.91–1.08 in a fixed-effect meta-analysis under a dominance model where the effect is due to the presence of the antigen) or in any individual population (e.g., OR = 1.02, 95% CI = 0.89–1.18 in the Gambia, Supplementary Fig. 7).

We considered whether imperfect imputation could explain the lack of observed association with HLA alleles. Imputed HLA antigen frequencies were broadly consistent with published frequencies estimated by serotyping in the same populations (Fig. 6b) and HLA-B*53 was imputed at a reasonably high frequency and high confidence (estimated frequency = 7–18%, IMPUTE info score > 0.94 in all African populations). To directly test imputation accuracy, we obtained HLA types of a subset of 31 Gambian case individuals by Sanger sequencing (Supplementary Data 5) and confirmed that HLA-B*53 was relatively well imputed in these samples (e.g., correlation = 0.92 between imputed dosage and true number of copies of the allele, reflecting five individuals imputed with probability >0.75 as heterozygote carriers of B*53, of which one appears incorrect). However, the closely related B*35 allele was much less accurately imputed, with four of eight carriers imputed as having non-B*35 genotypes, including one imputed to carry B*53. This may be the reason for the relatively low observed frequency of imputed B*35 alleles (Fig. 6b) and is notable because B*53 is thought to have arisen from B*35 via a gene conversion^52^. Thus, although our failure to replicate this widely cited association appears robust, future work to improve HLA inference in these populations is needed to provide additional clarity.

### Allele frequencies suggest polygenic positive selection

**Fig. 7:**
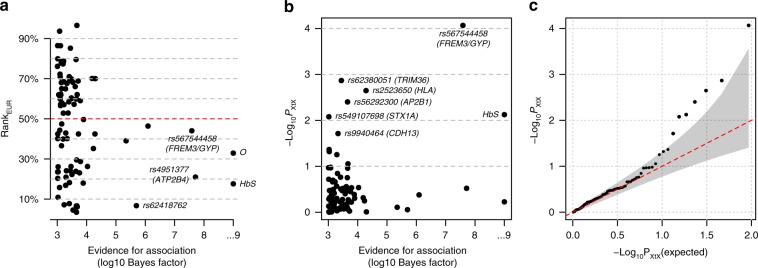
Empirical evidence for frequency differentiation of the most associated alleles. **a** The European population rank (rank_EUR_, y axis) plotted against the evidence for association (log10 BF_avg_, x axis) for the protective allele at each of the 91 lead variants satisfying BF_avg_ > 1000 and having assigned ancestral allele. For each variant with an estimated protective (respectively risk) derived allele *A*, rank_EUR_ is defined as the proportion of alleles genome-wide having lower or equal (respectively greater than or equal) count than *A* in European populations, conditional on having the same frequency in African populations, estimated in reference panel populations. On average, rank_EUR_ is expected to be equal to 50% (red dashed line). Points are labelled by the rsid and nearest or relevant gene(s), or by functional variant where known; O refers to rs8176719, which determines the O blood group. **b** Evidence for within-Africa differentiation (*P*_XtX,_ y axis) plotted against the evidence for association (log10 BF_avg_, x axis) for each of the 92 lead variants satisfying BF_avg_ > 1000, after removing all but rs2523650 from within the HLA region. *P*_XtX_ is computed from an empirical null distribution of allele frequencies learnt across control samples in the seven largest African populations (Supplementary Figs. 8 and 9). **c** Quantile–quantile plot for *P*_XtX_ across the top 92 regions in **b**. Source data are provided as a Source Data file.

Malaria is regarded as having played a strong role in shaping the human genome through natural selection^53,54^ and observations of allele frequency differences are commonly used to motivate the study of specific mutations (e.g., see refs. ^55–57^). We sought to assess whether variants associated with malaria susceptibility show evidence of selection by comparing with genome-wide allele frequency variation between populations (Fig. 7 and Supplementary Figs. 8–10). We found that the alleles with the strongest evidence of protection tend to be at a lower frequency in non-African populations than expected, given the genome-wide distribution (e.g., conditional rank of allele in European populations given African allele count (rank_EUR_) < 0.5 for 10 of 12 regions with BF_avg_ *>* 10,000; 45 of 91 regions with BF_avg_ *>* 1,000; based on reference panel allele counts; Fig. 7a), consistent with the hypothesis that these alleles have been maintained at a high frequency in African populations by positive selection. However, this comparison provides extremely modest levels of evidence—even for individual alleles where selection of this type is well accepted. We similarly found modest evidence for within-Africa differentiation (e.g., *P* = 3 × 10^−3^ against the null model of no differentiation, for the 12 regions with BF_avg_ > 10,000; *P* = 0.02 across 92 regions with BF_avg_ > 1000, excluding all but one variant within the HLA region; Fig. 7b, c). A further discussion of differentiated loci is found in Supplementary Note 9.

These analyses add new weight to the hypothesis that malaria-driven selection has played a polygenic role in shaping human genomes in endemic populations. However, this evidence is rather weak and it is equally clear that many of the most differentiated alleles, including those within the HLA (e.g., HLA-DPB*01; frequency range 25–54% in African populations; *P*_XtX_ = 8.9 × 10^−15^), variants in *CD36* (e.g., rs73711929, frequency range 2–20% in African populations; *P*_XtX_ = 8.3 × 10^−20^), as well as variants in regions associated with skin pigmentation^58^ (e.g., rs1426654; frequency range 83–99% in African populations; *P*_XtX_ = 2.5 × 10^−15^) are not associated with susceptibility to *P. falciparum* malaria (Supplementary Fig. 11). Other selective forces may have contributed to the evolution of these alleles^59^ and in many cases these remain to be identified.

## Discussion

This analysis identifies five replicable loci with strong evidence for association with resistance to SM: *HBB*, *ABO*, *ATP2B4*, the glycophorin region on chromosome 4 and a new locus between *MAP3K7* and *EPHA7* on chromosome 6 whose causal mechanism is unknown. The *HBB*, *ABO* and glycophorin associations correspond to known phenotypic variants of human RBCs, namely sickle cell trait and the O and Dantu blood group phenotypes. Adding to previous work^32^, our analysis indicates that the associated haplotype in *ATP2B4* also has a specific effect on erythrocyte function. The putative protective allele disrupts a GATA1-binding site upstream of a TSS, which is only active in erythroid cells, and thus reduces expression of this gene in erythrocyte precursors. This is reminiscent of the protective effect of the Duffy-null allele against *P. vivax* malaria, which also involves disruption of a GATA1-binding site causing erythrocyte-specific suppression of the Duffy antigen receptor^46,47^, and it adds to growing evidence that tissue-specific transcriptional initiation is an important factor in determining human phenotypes^60,61^. *ATP2B4* encodes the main erythrocyte calcium exporter and the associated haplotype has been experimentally shown to control calcium efflux and hydration^32,40^. This is consistent with the observed effects on red cell traits^36^ and with the hypothesis that *ATP2B4* modulates parasite growth within erythrocytes, perhaps by affecting calcium signalling mechanisms that are essential to several stages of the parasite life cycle^62,63^. In theory, this finding raises the possibility of therapeutic blockage of *ATP2B4* in erythrocytes without otherwise affecting physiology. Based on the estimated effect and population frequency of the associated haplotype, this might be expected to provide ~1.5-fold protection against SM and be efficacious in the ~90% of individuals who do not currently carry the protective homozygous genotype at this locus. It is open to speculation whether this could lead to worthwhile therapy in practice.

The five loci identified so far appear to explain around 11% of the total genetic contribution to variation in malaria susceptibility (Supplementary Fig. 4). It is possible that this estimate is confounded, e.g., by unusual LD patterns or by environmental factors such as variation in underlying infection rates, and it should likely be treated with caution. Nevertheless, it raises the question as to why only five loci can be reliably detected at present. Selection due to malaria has been sufficiently strong to maintain alleles such as sickle haemoglobin at high frequency in affected African populations^64^ (Fig. 7). However, it has evidently also led to complex patterns of variation at malaria resistance loci. This is exemplified by the emergence of haemoglobin C and multiple haplotypes carrying sickle haemoglobin at *HBB*, and by copy number variation observed at the alpha globin and glycophorin loci^14,65^. In addition, there is evidence for long-term balancing selection at the ABO^66^ and glycophorin^67^ loci, which may be malaria-related. In theory, the simplest outcome for a strongly protective allele is that it would sweep to fixation, but unlike for *P.vivax*^68^ no sweep of a mutation providing resistance to *P. falciparum* malaria is known. Remaining protective alleles may therefore have effect sizes too small to be subject to strong selection, requiring large sample sizes to detect. GWASs of other common diseases have shown the benefit of exceptionally large sample sizes^69–73^ and, although there are many practical obstacles to achieving this for SM, particularly as its incidence has fallen in recent years due to improved control measures^74,75^, efforts to collect new samples and to combine data across studies^63^ are warranted. It is also possible that relevant alleles may have become balanced due to pleiotropic effects, or be subject to compensatory parasite adaptation—both scenarios that might lead to maintenance of allelic diversity. Dissecting such signals is naturally challenging for GWAS approaches and may require new techniques, such as sequencing and joint analysis of host and parasite genomes from infected individuals, to address.

## Methods

### Ethics and consent

Sample collection and study design was approved by Oxford University Tropical Research Ethics committee (OXTREC), Oxford, UK (OXTREC 020–006). Local approving bodies are detailed in Supplementary Table 1. Further information on policies, research and the consent process may be found on the MalariaGEN website (http://www.malariagen.net/community/ethics-governance).

### Collection and processing of whole-genome sequence data

Blood samples were collected from a total of 773 individuals from Gambia, Burkina Faso, Cameroon and Tanzania, and sequenced to an average of 10× coverage on the Illumina HiSeq 2000 platform at the Wellcome Sanger Institute, using 100 bp paired-end reads. In addition, one Gambian trio was additionally sequenced using three alternate library preparation methods (low-quantity and whole-genome amplification pipelines) for a total of 782 sequenced samples. Sequence reads were mapped to the GRCh37 human reference genome with additional sequences as modified by the 1000 Genomes Project (hs37d5.fa), using BWA^76^ with base quality score recalibration and local realignment around known indels as implemented in GATK^77^.

We used GATK UnifiedGenotyper to identify potential polymorphic sites on autosomal chromosomes, using all 782 samples as well as the 680 samples of African ancestry from the 1000 Genomes Project, and running in 50 kb chunks. GATK VQSR was used to separately filter SNPs and indels. For SNPs, we included three training sets: HapMap SNPs, SNPs on the Omni 2.5 M array and 1000 Genomes Phase 3 sites. For indels we used the ‘Mills’ training set as recommended in the GATK documentation. We based filtering on read depth, mapping quality, quality by depth, the MQRankSum and ReadPosRankSum measures of bias in reference vs. alternate allele-mapping quality or read positions, and tests for strand bias and inbreeding coefficient. We filtered variants applying a sensitivity setting of 99.5% (--ts_filter_level 0.95) for SNPs and 95% for INDELs (--ts_filter_level 0.95). We further restricted to biallelic sites.

We computed genotype likelihoods at all sites passing the filter described above and sites in 1000 Genomes Project Phase 3 reference panel using GATK HaplotypeCaller. We then used BEAGLE v4.0^78^ to generate genotype calls from the genotype likelihoods. We ran BEAGLE in windows of 50,000 SNPs, including trio information for Gambian samples. Due to the observed long runtimes when sampling trios, we specified trioscale = 3 when running BEAGLE to reduce computation times. We then concatenated results across chromosomes.

To estimate haplotypes, we first removed variants not present in the 1000 Genomes Phase 3 panel or variants that had alleles differing from those in the 1000 Genomes Phase 3 panel, using the ‘check’ mode of SHAPEIT2^79^. We then used SHAPEIT2 to phase each chromosome. We included trio information for Gambian samples, specified a window size of 0.5 (--window), an effective sample size of 17,469 (--effective-size), 200 model states (--states), included 1000 Genomes Phase 3 haplotypes in the phasing process (--input-ref) and used the hapmap-combined recombination map in build 37 coordinates (-M).

To construct a combined imputation reference panel, we first removed repeated samples and then combined phased haplotypes at the remaining samples with those from the 1000 Genomes Project phase 3 panel. The final panel contains 77,931,101 variants and 3277 samples, of which 773 are from our set of sequence data and 2504 are from the 1000 Genomes panel. In total, the panel includes 1434 individuals with recent African ancestry (including 157 African American samples) and non-African samples as previously described^11^.

### Collection and genotyping of GWAS data

Cases of SM were recruited on admission to hospital as part of ongoing studies by local investigators (Supplementary Table 1). SM phenotypes were ascertained in a standardized manner across all samples using definitions as per WHO guidelines^23^ for CM (Blantyre coma score < 3 in children or Glasgow coma score  < 11 in adults), SMA (haemoglobin < 5 g/100 mL or haematocrit < 15%) and other malaria-related symptoms (here referred to as ‘other SM’). The distribution of these phenotypes between countries is summarized in Supplementary Fig. 5. Control samples representative of the ethnic groups of the cases were collected from cord blood or in some study sites from samples from the local population^5^. For our main discovery and replication analyses, we maximized sample size by including all samples collected as cases and controls. For specific estimation of effect sizes (Fig. 4), we restricted to a ‘strict’ definition of case status defined as having positive measured parasitaemia. Further details of this study have been published previously^5^ and information on policies, research and the consent process may be found on the MalariaGEN website (http://www.malariagen.net/community/ethics-governance).

Sample genotyping was performed at the Wellcome Sanger Institute using the Illumina Omni 2.5 M platform. Genotype calling was performed using three genotype calling algorithms (Illumina Gencall, GenoSNP^80^ and Illuminus^81^) and we formed final genotype calls by taking the consensus of the three algorithms, or of two algorithms where the third algorithm reported a missing call, as used previously^7^. Genotypes for which a discrepant call were made were treated as missing.

We used strand information from array manifest files provided by Illumina and those from a remapping process implemented by William Rayner at the Wellcome Centre for Human Genetics, Oxford, to determine the strand of each assay on each of the two genotyping platforms used. We omitted variants where mapping or strand information was discrepant, with some adjustments for Omni 2.5 M ‘quad’ array as described below. In addition, we annotated each variant with the reference allele as taken from the build 37 reference sequence FASTA file. We used these data with QCTOOL (see Code availability) to realign study genotypes. The resulting ‘aligned’ datasets are encoded so that the first allele reflects the reference allele, the second allele the non-reference allele and all alleles are expressed relative to the forward strand of the reference sequence, simplifying downstream analysis.

As a sanity check, we plotted the estimated frequency of each non-reference allele against the frequency in the closest reference panel group in each populations. The Kenyan dataset (which was typed using the Omni 2.5 M quad ‘D’ version manifest) showed a substantial number of SNPs with frequencies obviously wrongly specified. Comparison of the flanking sequence and alleles reported in the manifest file identified 18,763 SNPs that had alleles coded incorrectly in the manifest file. We repaired fixed these SNPs by recoding these alleles, making them consistent with the flanking sequence, the more recent ‘H’ version of the manifest, and the Omni 2.5 M ‘octo’ chip manifest. We also updated the positions of 495 SNPs annotated as lying on the pseudo-autosomal part of the X chromosome (Chr = ‘XY’ in the Illumina manifest) but with position = 0. We applied these updates, repeated the alignment process for Kenya, and regenerated the frequency plot. A small number of SNPs in each population still show distinct frequencies compared with reference panel data (Supplementary Fig. 13).

### Sample quality control

We used QCTOOL to compute the proportion of missing genotype calls and the average proportion of heterozygous calls per sample, and the average X channel and Y channel intensities across autosomal chromosomes for each sample. Values were averaged over a subset of SNPs (‘the quality control (QC) SNP set’) chosen to have low missing rates in all populations. We then ran ABERRANT^82^ to identify individuals with outlying mean intensities separately in each population. Considerable variation in the spread of quality between populations was observed and we picked population-specific values of the ABERRANT lambda parameter based on visual inspection of the plots. We also manually adjusted the ABERRANT-derived exclusions to include a small number of samples in each population, which were observed to visually cluster with the main set of included samples, but called as outlying by ABERRANT. We then plotted logit (missing call rate) against average heterozygosity in each population. We excluded samples with >10% missingness or heterozygosity outside the range 0.2–0.4 in African populations, below 0.175 in Vietnam or below 0.13 in PNG outright computed the QC SNP set. We then applied ABERRANT to the remaining non-excluded samples choosing a value of the lambda parameter per population based on inspection. As for intensity outliers, we specifically included some groups of samples visually clustering with the main group of samples but called as outlier by ABERRANT. We plotted heterozygosity against missingness annotating the removed samples. Supplementary Figs. 14 and 15 show mean intensity, missingness, and heterozygosity, computed across all variants, with excluded samples annotated.

The above process results in a set of 18,515 samples that appear to be relatively well genotyped across the autosomes. In addition, for some purposes described below, we used a smaller set 17,664 samples (referred to as the ‘conservative’ set of samples) obtained by repeating the above process with more conservative values of the ABERRANT lambda parameter.

Almost all samples had sex determined by direct genotyping of amelogenin SNPs^5^. To confirm the assigned sex, we plotted overall intensity from the Illumina chip (X channel plus Y channel intensity) averaged across the X chromosome, against overall intensity averaged across the Y chromosome (Supplementary Fig. 16). We then modelled these mean intensities as drawn from a bivariate normal distribution, with separate mean and covariance parameters in males and females. A small number of samples were observed to lie near a point consistent with female-like intensity values on the X chromosome but male-like intensity values on the Y chromosome; this could potentially indicate males with an extra copy of the X chromosome and we therefore further included an additional ‘mixed’ cluster with parameters derived from the male and female clusters on the X and Y chromosome, respectively, and assuming no covariance between X and Y chromosome intensities. We used the amelogenin SNP-based assignments and the intensities for the conservative samples set to estimate the model parameters. Based on the fitted model we then probabilistically assigned sex to all samples using Bayes rule:

1 $$P\left( {{\mathrm{sex|intensities}}} \right) \propto P\left( {{\mathrm{intensities|sex}}} \right) \times P\left( {{\mathrm{sex}}} \right)$$

where the first term on the right is computed using the bivariate normal model described above and the second represents a prior probability on the sample sex. We normalize the formula under the assumption that every sample is either male, female or ‘mixed’. We specified a 1% prior probability of ‘mixed’ sex and equal prior probability of 49.5% on each of the male and female clusters. Finally, we assigned each sex having at least 70% posterior probability and treated the sex of other samples as uncalled. Of the 18,515 samples in the QC set, 20 had mixed or missing sex by this assignment, whereas 38 had assignment discrepant with the amelogenin-determined sex. We removed samples with mixed or missing sex from downstream analyses.

To identify relationships between samples, we used QCTOOL to compute a matrix of pairwise relatedness coefficients in each population. To do this, we first chose a subset of SNPs using a preliminary version of the SNP QC process described below and used inthinnerator (see Code availability) to thin this set of SNPs to lie no closer than 0.02 cM apart in the Hapmap-combined recombination map. We additionally excluded SNPs from regions of known associations, the region chr6:25,000,000–40,000,000, which contains the HLA, and the region of the common inversion on chromosome 8. We used QCTOOL with the resulting set of 157,085 SNPs to compute the normalized allele sharing (‘relatedness’) matrix *R* *=* *(r*_*ij*_*)* where *r*_*ij*_ reflects the correlation in genotype between sample *i* and *j*, normalized by variant frequency. The number *r*_*ij*_ may be treated as an estimate of relatedness with values near 1 reflecting identity and values near 0 reflecting unrelated samples, relative to the average relatedness in the population^83,84^.

For each pair of samples with relatedness > 0.75, we noted the sample with the highest rate of missing genotype calls. A total of 538 samples were identified in this way, the majority of which were from the Malawi and Kenya datasets, and likely reflect duplicate genotyping. We removed these samples from the datasets. For each remaining pair of samples with relatedness > 0.2, we again noted the sample with the highest rate of missing genotype calls; these represent samples that are relatively closely related to other samples in the same study sample. A total of 801 samples were identified in this way. These samples were retained in the dataset for phasing and imputation, but excluded from PC analysis and downstream association testing.

We computed PCs by taking the eigendecomposition of *R* and plotted PCs in each population. Based on visual inspection of these plots, we further identified a total of 40 samples outlying on any of the first 10 PCs. We recomputed *R* and the PCs after excluding these individuals, to produce a set of PCs for association analysis. PCs were observed to correlate with population structure (as represented by the reported ethnicity) and in some populations with case/control status and/or with technical factors such as missing genotype call rates.

In total, following our sample QC process, 17,960 samples passed QC criteria, had non-missing/non-mixed sex assignment and were not identified as duplicates as described above. We took all of these samples through to the phasing and imputation as described below. Of these samples, a total of 17,056 samples were identified as not outlying on PCs, not closely related and having assigned case/control status, and were included in downstream analyses. Supplementary Table 3 summarizes the sample QC.

### SNP quality control

We observed study sample sets to vary considerably in quality of typing (as well as in size), with the two smallest African sets (Mali and Nigeria) and, to a lesser extent, Malawi having higher missing data rates and Kenya having lower rates (Supplementary Fig. 17). Inspection of cluster plots suggests that the higher missing rates are largely due to higher levels of noise in intensity values for these populations.

We based SNP QC on several metrics computed separately in each population: the estimated minor allele frequency (MAF), the proportion of missing genotype calls (missingness), a *P*-value for Hardy–Weinberg equilibrium (HWE)^85^ computed in control samples (*P*_HWE_), a plate test *P*-value against the null model that genotypes are uncorrelated with the plate on which each sample was genotyped (*P*_plate_) and a recall test *P*-value, which compares the genotype frequencies with frequencies after a re-calling process based on the estimated cluster positions across populations. The plate test was implemented using a logistic regression model treating the genotype as outcome and an indicator of the 96-well plate on which the sample was supplied for genotyping as a predictor in each population, and including case/control status and an initial set of five PCs as covariates. For each SNP we computed a likelihood ratio test *P*-value for the inclusion of plate as a predictor. The recall test was motivated by the observation that some otherwise well-typed SNPs were poorly genotyped in individual populations and was implemented as follows. We first used intensities and genotype calls for the ‘conservative’ sample set to re-estimate the positions of genotype clusters at each SNP in each study population using QCTOOL, fitting a multivariate *t*-distribution with 5 degrees of freedom to each genotype cluster. For each SNP in each target population, we then re-called genotypes using intensities based on a mixture of the cluster positions learned in all other populations. We used Fisher’s exact test to compute a *p*-value against the null that the frequency in the original and re-called genotypes were the same.

We used association test statistics computed under a general logistic regression model, including additive and heterozygote parameters, as a guide to choosing appropriate thresholds for the above metrics. Specifically, we tested for association with SM, including five PCs as covariates in each population. For the given QC criteria, we plotted association test results annotating QC fails, along with qq plots for the variants passing QC. In choosing thresholds, we were motivated by the observation that the combination of consensus genotype calling (which is relatively conservative in calling genotypes) with statistical phasing across populations would likely produce a high-quality set of haplotypes for downstream inference. We therefore aimed to produce a single set of SNPs with high-quality data across populations for input to the phasing process. We chose a set of criteria applied to the nine largest sample sets to compute a list of SNPs to include. We removed SNPs with missingness >2.5% in Kenya and PNG, >5% in Gambia, Burkina Faso, Ghana, Cameroon and Vietnam, and >10% in Malawi and Tanzania. We also excluded SNPs with *P* < 1 × 10^−20^ for HWE, *P* < 1 × 10^−3^ for the plate test and *P* < 1 × 10^−6^ for the recall test in each population. Finally, we included each of the remaining SNPs that was at MAF > 1% in at least two populations and passed the above thresholds in all nine populations considered. Data from the Mali and Nigeria populations, which had the lowest sample counts (484 and 133 samples, respectively) and empirically lower rates of high-quality genotyping (Supplementary Fig. 17), was not used to inform the SNP selection process. The criteria above were observed to lead to essentially uninflated association test statistics (Supplementary Fig. 18). In particular, we noted that a set of SNPs close to the ends of chromosomes were excluded by these criteria; more information on this is provided in Supplementary Note 2 and Supplementary Fig. 19.

SNP QC on the X chromosome was performed as for autosomes with the following adjustments. We excluded the pseudo-autosomal region (defined as variants with position <2,699,520 or >154,931,044; this region contained ~400 typed SNPs). We also observed elevated rates of males called as heterozygous within the X transposed region (defined as chrX:88,457,462–92,374,313) and excluded this region as well. Approximately 3400 SNPs were removed this way. In computing summary statistics for males, we treated all heterozygous calls as missing and homozygous calls as the corresponding hemizygous calls. We applied the SNP missingness threshold and the plate test separately in males and females; we replaced the HWE test with a test of difference in frequency between males and females (based on a likelihood ratio test *P* < 1 × 10^−6^). We did not implement the recall test for X chromosome SNPs.

Finally, we plotted cluster plots for all SNPs with genotypic association test *P* < 1 × 10^−5^ and manually excluded those with obviously problematic cluster plots. Some SNPs are targeted by duplicate assays on the Illumina Omni 2.5 M array and for these SNPs we excluded the assay showing the highest missingness, on average, across populations. We also removed SNPs present on only one of the ‘quad’ and ‘octo’ platforms. Finally, we merged data for SNPs passing QC across populations using QCTOOL for input to phasing. Supplementary Table 4 summarizes the number of SNPs excluded by each criteria and the final QC set.

### Phasing and imputation

We used SHAPEIT2^79^ to jointly phase the 17,960 samples passing QC. In detail, we ran SHAPEIT2 on each chromosome separately specifying an effective sample size of 17,469 and 200 copying states, and using the HapMap combined recombination map. As above, we note that in addition to phasing, SHAPEIT2 imputes missing genotypes at typed SNPs with missing data, so that the output of this step contains hard-called phased genotypes with no missing data. Following phasing we recomputed PCs across all populations, across African populations, and in each population separately, using phased genotypes. These PCs are shown in Fig. 1 and Supplementary Fig. 2.

We used IMPUTE2 (v2.3.2)^86^ to impute genotypes at all variants present in the combined reference panel. To do this efficiently, we split the data into subsets of 500 samples and split the genome into a total of 1456 chunks of 2 Mb each. We ran IMPUTE2 in each chunk and sample subset specifying a buffer region of 500 kb and an effective population size of 20,000. Finally, we used QCTOOL to merge imputation chunks across sample subsets and across chunks, encoding the results in BGEN format^87^. For post-imputation analysis, we additionally merged all impute ‘info’ files across chunks and sample subsets into a single file. We also repeated imputation using the 1000 Genomes Phase III reference panel (as available from the IMPUTE2 website 3 August 2015) using the same settings throughout.

To assess per-variant imputation performance, we focused on ‘type 0 *r*^2^’ (which measures correlation between input genotypes and re-imputed genotype dosages), which we refer to here as accuracy. We plotted mean accuracy in 1% MAF bins for each subset of samples imputed (Supplementary Fig. 1). We also plotted the proportion of variants meeting a given accuracy threshold for lower-frequency (<10% MAF) and higher-frequency (10–50% MAF) variants. Under the assumption that untyped variants behave similar to typed variants, these plots suggest that a substantial proportion of variation in the genome is imputed to high accuracy using this panel. We additionally computed the mean difference in accuracy, within MAF bins, for each imputation sample subset (Supplementary Fig. 1). We used the per-sample imputation results output by IMPUTE2 to compare imputation performance across ethnic groups. IMPUTE2 outputs an accuracy measure (also called type 0 *r*^2^, which we refer to as per-sample accuracy) for each sample for each imputation chunk, reflecting the correlation between input genotypes and re-imputed genotype dosages across variants within each chunk. We plotted the distribution of per-sample accuracy, averaged across chunks, for samples in each of the largest ethnic groups in our data (Fig. 1).

To further investigate the effects of additional African reference panel samples on imputation performance, we constructed a joint dataset consisting of phased haplotypes at all 17,960 study individuals and all 3046 reference panel individuals, subsetted to the overlapping set of 1,492,601 SNPs. For each pair (S,P) of a study panel haplotype S and a reference panel population P, we computed the proportion of 1 Mb chunks such that the closest reference panel haplotype to S lies in P. Proximity was measured by absolute number of differences with ties broken by randomly choosing one of the closest haplotypes. We averaged the proportions over samples in each study ethnicity; these proportions are plotted in Fig. 1c.

### Imputation of HLA and glycophorin alleles

We used HLA*IMP:02^50^ to impute HLA classical alleles for all study samples. We used the unphased, post-QC set of genotype calls across populations in the region chr6:28Mb-36Mb as input. This version of HLA*IMP outputs imputed diploid allele calls and posterior probabilities for alleles at two-digit and four-digit resolution, and uses an allele naming scheme that is similar to the pre-2010 IPD-IMGT/HLA naming convention. For downstream analysis, we split HLA*IMP output into per-allele posterior probabilities. Specifically, for each allele we computed the posterior probability of zero, one or two copies of the allele, against all other alleles at the locus, from the HLA*IMP output. We encoded this data in the GP field of a VCF file with one row per allele. This functionality may be generally useful and is implemented in QCTOOL. For reference purposes, we assigned each allele to the midpoint of the corresponding gene.

We used IMPUTE2 to impute genotypes from a previously published reference panel of SNPs, indels and large copy number variants (CNVs) in the glycophorin region on chromosome 4^14^. We based our imputation on the phased set of haplotypes, but to avoid potential issues with phasing in this region we treated these genotypes as unphased. We then ran IMPUTE2 in 2 Mb chunks across a 10 Mb region surrounding the glycophorins, specifying 1000 reference panel haplotypes, a 500 kb buffer region, an effective population size of 20,000, and using the HapMap combined recombination map. As previously^14^, we excluded SNPs in the region of segmental duplication when imputing CNV calls.

### Association testing

We used SNPTEST (see Code availability) to test each typed or imputed variant for association with SM status in each study population. Specifically, for each population we ran SNPTEST for each of the 2 Mb chunks output by imputation, including either 0, 2, 5 or 10 population-specific PCs as covariates and under additive, dominant, recessive, heterozygote and general models of association. For the main analyses presented here, we used the versions based on including five PCs. A total of 17,056 samples had PCs and assigned case/control status, and were included in association analyses. We tested under additive, dominant, recessive and heterozygote inheritance models.

To test association with the main SM subphenotypes in our data—namely CM and SMA, and other SM cases (OTHER)—we extended SNPTEST to perform maximum likelihood inference for multinomial logistic regression. A full description of this method is presented in Supplementary Note 6. In brief, in this framework, the likelihood is parameterized by the log odds of each case phenotype (CM, SMA or OTHER) relative to the baseline phenotype (CONTROL), for the genotypic predictor along with other covariates. The method produces maximum likelihood estimates of these parameters and estimated parameter SEs and covariances. Overall evidence for association can then be assessed by computing a likelihood ratio test statistic and associated *P*-value, against the null model where all genetic effect-size parameters are zero. In addition, for each case phenotype we compute a Wald test statistic and *P*-value against the null model that the genetic effect on that phenotype is zero. Of the 17,056 samples included in association testing, 537 had no subphenotype assignment or were assigned as having both CM and SMA phenotypes. For simplicity we excluded these samples from the genome-wide test against subphenotypes. We tested under additive, dominant, recessive and heterozygote inheritance models.

We additionally tested variants on the X chromosome using a logistic regression model including sex as a covariate and treating male hemizygote genotypes in the same way as homozygous females.

### Genome-wide meta-analysis

To efficiently meta-analyse the association results, we further developed our software package BINGWA^7^ (see Code availability). BINGWA is written in C++ and takes a list of SNPTEST files, along with options that control variant filtering and output, and computes meta-analysis results using both frequentist and Bayesian methods. Per-population results for each variant were included in meta-analysis if the minor allele count (or for non-additive tests, the minor predictor count as defined below) was at least 25, the IMPUTE info was at least 0.3 and no issues were reported with model fitting. For the non-additive tests, we defined the minor predictor count as the minimum of the expected number of individuals having the effect genotype (e.g., AB or BB for dominant model of the B allele, BB for recessive model, etc.) and the number having the baseline genotype (e.g., AA for a dominant model of the B allele, AA or AB for recessive model, etc).

For each case/control or subphenotype test, we computed a frequentist fixed-effect meta-analysis estimate **β**_meta_, its variance–covariance matrix **V**_meta_ and overall meta-analysis *P*-value as described in Supplementary Note 7. For subphenotype tests, **β**_meta_ is three dimensional (corresponding to joint estimates for CM, SMA and other severe malaria cases); we additionally computed a Wald test *P*-value for each estimated parameter.

To assess evidence for association under a more flexible set of models of association, we used a Bayesian meta-analysis framework similar to that described previously^5,7,12^. In brief, we implemented Bayesian inference using the asymptotic or approximate Bayes factor approach^88^. This approach treats the observed effect sizes (estimated by logistic or multinomial logistic regression in each population) as arising from a set of true population effect sizes, together with estimation noise that is represented by the SEs and parameter covariances in each population. The ‘true’ population effects are modelled as being drawn from a multivariate normal distribution with mean zero and a prior covariance matrix Σ, which is chosen to represent a desired model of true effects. We write this covariance in the form

2 $$\Sigma = \sigma {{\mathbf{P}}}\sigma$$

where *Ρ* is a correlation matrix specifying the prior correlation in true effect sizes between populations and/or between subphenotypes, and *σ* is a scalar (or, in the general case, a diagonal matrix), which determines the prior variance of effect sizes, and thus the overall magnitude of modelled true effects.

Given the vector of per-population estimates **β** and the parameter covariance matrix **V**, a Bayes factor for the model encoded by **Σ** can now be computed as a ratio of multivariate normal densities

3 $${\mathrm{BF}} = \frac{{\mathrm{MVN}\left( {{\mathbf{\upbeta }};0,\mathbf{V} + \mathbf{\Sigma}}\right) }}{{\mathrm{MVN}\left( {{\mathbf{\upbeta }};0,{\mathbf{V}}} \right)}}$$

Different choices of Σ correspond to different assumptions about the underlying true effect sizes. The true pattern of effects is unknown, so we assess evidence under a collection of plausible assumptions Σ_1_, Σ_2_, … by computing the model-averaged Bayes factor

4 $${\mathrm{BF}}_{{\mathrm{avg}}} = {w_1}{\mathrm{BF}}_1 + {w_2}{\mathrm{BF}}_2 + \ldots$$

in which BF_*i*_ is the BF computed using prior covariance **Σ**_**i**_ and the prior weights *w*_*i*_ are chosen to sum to 1. The specific choices of prior correlation matrix **Ρ** and weights *w* used for our primary analysis are described below and in Supplementary Table 5. For each choice of correlation matrix **Ρ**, we computed the BF for that model as an equally weighted average over four values of **σ**—namely 0.2, 0.4, 0.6 and 0.8. We also repeated all analyses under additive, dominant, recessive and heterozygote modes of inheritance with prior weights 0.4, 0.2, 0.2 and 0.2, respectively.

For case/control tests we considered the following models of effects across all populations:

- fixed effects (all off-diagonal entries of **Ρ** set to 0.99)
- correlated effects (all off-diagonal entries of **Ρ** set to 0.9)
- independent effects (all off-diagonal entries of **Ρ** set to 0)
- structured effects (**Ρ** estimated from the correlation in allele frequencies genome-wide)

We placed a total of 60% prior weight on fixed and correlated effects and 4% prior weight on each of independent and structured effects. In addition to effects across all populations, we included models of effect restricted to population subgroups. Specifically, we focused on the following groupings:

- West African populations (Gambia and Mali, or Gambia, Mali, Burkina Faso and Ghana)
- West and Central West African populations (Gambia, Mali, Burkina Faso, Ghana, Nigeria and Cameroon)
- Central West African populations (Burkina Faso, Ghana, Nigeria and Cameroon)
- Central and East African populations (Nigeria, Cameroon, Malawi, Tanzania and Kenya)
- East African populations (Malawi, Tanzania and Kenya)
- All African populations (Gambia, Mali, Burkina Faso, Ghana, Nigeria, Cameroon, Malawi, Tanzania and Kenya)
- Non-African populations (Vietnam, PNG)

Population subset models were implemented by setting the value of **σ** to 0.001 for non-associated populations. These subsets are chosen to correspond to geographical groupings, as well as to groups apparent along the first PC of African populations (Fig. 1e), and such that no group has fewer than 2000 samples. We placed 4% prior weight on each of the eight groups, for a total weight of 1 (Supplementary Table 5).

In our results files and in Supplementary Data 1, these population groupings are denoted by a string of eleven 0s and 1s, with populations ordered west–east as in Fig. 1a, and a 1 indicating that the effect is assumed nonzero in the corresponding population. For example, the model fix:11111111100 denotes fixed effects across all African populations.

We placed 80% of prior mass on case/control effects as described above, but also considered effects that vary across subphenotypes (CM, SMA and other SM), as well as effects that are only present for only two or one of the subphenotypes (Supplementary Table 5). Specifically, we considered:

- Effects on all three subphenotypes (between-phenotype entries of *Ρ* set to 0.9 or 0).
- Effects on two of three subphenotypes (*σ* set to 0 for one phenotype, between-phenotype entries of *Ρ* set to 0.9 or 0).
- Effects restricted to one subphenotype (*σ* set to 0 for two phenotypes)

We assigned these categories prior weights of 8%, 6% and 6%, respectively. To avoid spurious results, when conducting subphenotype meta-analysis we assumed that effects were fixed across populations; specifically, between-population within-phenotype entries of *Ρ* were set to 0.99 and between-population between-phenotype entries of *Ρ* were set to 0.99 times the assumed between- phenotype correlation specified above. The results of using the set of models and prior weights described above to compute BF_avg_ are shown in Fig. 2.

To allow further assessment of the dependency of BF_avg_ on the model assumptions, the full meta-analysis output produced by BINGWA includes

- A full set of BFs, computed under a larger set of models, including those making up BF_avg_.
- The model with the highest BF.
- The model with the highest posterior weight (i.e., highest BF after the weighting) and the model with second highest posterior weight.
- A per-population BF reflecting the evidence from each individual population (as for BF_avg_, these are computed by model averaging over parameters *σ* *=* 0.2, 0.4, 0.6, 0.8. For subphenotype tests we assume independence between effects in different subphenotypes).
- Meta-analysis effect-size estimates and SEs, computed under the frequentist fixed-effect model, with associated *P*-values.
- A summary of the set of populations and number of samples that were included in meta-analysis.
- Additional information, including genotype counts and INFO scores, taken from the SNPTEST result files.

In our implementation, BINGWA was used to store meta-analysis for each mode of inheritance in a sqlite database, with results indexed by variant position and identifiers, and we used sqlite features to construct the final meta-analysis.

### Interpretation of the Bayesian model average

Conditional on the set of prior assumptions outlined above, the Bayes factor can be interpreted directly in terms of a prior odds of association by the formula

5 $${\mathrm{Posterior}}\,{\mathrm{odds}} = {\mathrm{prior}}\,{\mathrm{odds}} \times {\mathrm{BF}}_{{\mathrm{avg}}}$$

In the absence of other information about a given genetic variant, plausible values of the prior odds might be in the range 10^−5^–10^−7^ as described previously^15,89^. For a Bayes factor of 1 × 10^5^, this would therefore give posterior odds of association between one and one-hundredth (i.e., posterior probability of 1–50%). This computation thus reflects our belief about the top signals in our data, namely, that there is overwhelmingly strong evidence for signals at *HBB*, *ABO*, *ATP2B4* and at the glycophorin region (for which BF_avg_ > 5 × 10^7^), good evidence at *ARL14* and rs62418762 (BF_avg_ > 5 × 10^6^) and some evidence at further loci among the top 12 (BF_avg_ > 1 × 10^5^). Except for the association at *ARL14*, these observations are further reinforced by the replication data (Fig. 2c).

Given the assumptions underlying BF_avg_, the relative evidence for different models can also be interpreted as in Fig. 2. For example, at rs8176719 since the Africa-only model has prior weight 0.04 × 0.8 = 0.032 (averaged over mode of inheritance), the observation of 90% posterior probability on an Africa-only model (Fig. 2) reflects the fact that Bayes factor for this model is around 280 times larger than under any other model. Specifically, under a dominance model of case/control effects, the computed Bayes factors are 4.5 × 10^17^ and 1.2 × 10^20^ for effects across all populations and across Africa only, a relative difference of 265. (The Bayes factor for fixed effects across Africa and Vietnam combined is 2.6 × 10^19^, although we did not include this model in BF_avg_).

The BF_avg_ gives a direct measure of evidence for association present in the data conditional on the assumptions. However, in the context of GWAS in which many variants are tested, it is also useful to understand the distribution of Bayes factors that would be observed by chance if no nonzero effect were present. A general way to do this is to treat the BF_avg_ as a test statistic and to compute or estimate its null distribution^90^. To do this for rs62418762, we adopted a simulation-based approach in which we first estimated the frequency of the three genotype classes in each population, across case and control samples. Next, we conducted multiple rounds of simulation. In each simulation we simulated genotype counts for controls and for CM, SMA, OTHER and CM + SMA cases in each population by sampling genotypes from a multinomial distribution as

6 $${\mathrm{Genotype}}\,{\mathrm{counts}}|{\mathrm{ phenotype}},\,{\mathrm{population}}\sim {\mathrm{multinomial(}}N,f{\mathrm{)}}$$

where *N* is the number of samples with the given phenotype in the population and **f** is the three-vector of estimated genotype frequencies for the three genotype classes in that population. Each simulation is thus performed by generating a 5 × 3 array of genotype counts in each population. We implemented the simulations in R. For each simulation we then used the vcd package^91^ to compute the log-odds ratios for all cases against controls and for each SM subphenotype against controls, for each mode of inheritance, along with SE and covariances. We passed these estimates into the meta-analysis pipeline described above for discovery data to compute BF_avg_. Similar to the discovery analysis, to exclude spurious results due to small counts, populations with minor allele count < 10 (for additive model tests) or minor predictor count < 10 (for non-additive model tests) were excluded from meta-analysis.

The procedure described above produces a simulated distribution of BF_avg_ under the null, conditional on observed genotype frequencies. In total, we conducted 712,650,010 rounds of simulation, of which 5 had BF_avg_ larger than the observed BF_avg_ for rs62418762. The distribution of resulting Bayes factors is presented in Fig. 3c.

### Bayesian replication analysis

The Bayesian framework outlined above naturally extends to a discovery/replication setting; this is described further below and in Supplementary Note 5.

### Selection and interpretation of candidate regions

To identify a set of variants with high-quality evidence, we first filtered variants based on minor allele count and imputation quality as follows. In each population, we computed an effective minor allele count (EMAC) by the formula

7 $${\mathrm{Effective}}\,{\mathrm{minor}}\,{\mathrm{allele}}\,{\mathrm{count}} = {\mathrm{IMPUTE}}\,{\mathrm{info}} \times {\mathrm{minor}}\,{\mathrm{allele}}\,{\mathrm{count}}$$

where the minor allele count refers to the number of the less frequent allele seen in that population. For imputed variants, this is the expected number given the imputed genotype distribution. We then formed an overall EMAC by summing this quantity across all 11 populations in our study (or, for variants where individual populations were omitted from meta-analysis, across all populations included in the meta-analysis). For downstream analyses, we considered all variants with EMAC ≥ 250. This is a relatively relaxed criterion: e.g., for a variant with IMPUTE info = 1 in each population, this corresponds to an overall MAF of 250/(2 × 17,056) = 0.75%. Using the combined genome-wide reference panel, a total of 26,035,208 variants passed this filter.

We ranked the filtered list of variants in decreasing order of BF_avg_ and used inthinnerator to define a set of ‘lead’ variants and association regions. Inthinnerator works by iteratively picking the variant with the highest rank as lead variant and excluding all other variants in a recombination interval around the chosen variant. We specified a recombination interval of 0.125 cM plus a margin of 25 kb on either side of each lead variant, with distances determined by interpolating the HapMap combined recombination map.

Association regions around *HBB* and around the glycophorin gene cluster on chromosome 4 are especially extensive. We treated these regions specially, defining them as the regions chr11:3.5 Mb–6.5 Mb and chr4:143.5 Mb–146 Mb, respectively, based on visual inspection of the association signal and we excluded these regions from the inthinnerator region definition process. Including these regions, a total of 97 association regions contained a variant with BF_avg_ > 1000 and are listed in Supplementary Data 1.

To visually inspect regions, we created a set of plots showing the evidence for association. Specifically, we created hit plots showing:

- The association signal (log10 BF_avg_), colouring points by LD with the lead variant computed using the African reference panel haplotypes and annotating variants that were directly typed and included in our phased set.
- Recombination rates from the HapMap combined recombination map.
- Regional genes and pseudogenes, taken from the UCSC Genome Browser refGene track downloaded on 9 June 2016.
- A set of annotations reflecting functional information (described further below).

To annotate variants with functional information, we ran Variant Effect Predictor^27^ from Ensembl tools release 75, to predict the functional consequence of each typed and imputed variants within each association region using the –everything flag. We additionally annotated variants with previously reported association for traits from the NHGRI/EBI GWAS catalog^36,38^ and a previously published GWAS of haematopoetic traits^35^, and with measured RNA expression levels (processed as described below), transcription factor-binding sites, chromatin state in erythrocyte precursors and other cells^29–31^, topologically associated domains^92^ and reported eQTLs across cell types^32–34^.

For each lead variant, we also created forest plots showing, for each mode of inheritance,

- the effect-size estimate and confidence interval estimated in each population
- the number of samples, variant frequency and IMPUTE info in each population
- the frequentist meta-analysis results
- a bar plot summarizing the Bayesian meta-analysis results

Forest plots for lead variants can be found in Supplementary Figs. 5 and 6. As described above, we also produced and inspected cluster plots of typed variants with evidence of association.

### Analysis of RNA-seq data from erythrocyte precursors

We obtained raw RNA-seq data from erythrocyte precursors^31,93^ from the Gene Expression Omnibus (GEO) using the sra toolkit. In total, data on eight cell types was processed: CD34+, BFU-E, CFU-E, early basophilic, late basophilic, proerythroblast, polychromatic and orthochromatic erythrocyte progenitor cells, with three replicates of each^31,93^. We also downloaded data on 12 fetal and 12 adult experimentally differentiated erythroblasts reported by Lessard et al.^32^. To process these data, we extracted FASTQ files from the SRA archives and mapped reads to the build 37 reference sequence using TopHat v2.0.14, using the Gencode v19 release of the human reference sequence and gene annotations. For the Lessard et al.^32^ data, we included the options --library-type fr-firststrand and --microexon-search to match the original processing. We also downloaded RNA-seq data from circulating erythrocytes^45^ from the GEO. These data were aligned to the build 37 reference sequence using bwa-mem^76^ and we marked duplicate reads using Picard (https://broadinstitute.github.io/picard).

To visualize coverage profiles across *ATP2B4*, we restricted attention to reads with mapping quality at least 50 and used bedtools genomecov^94^ to compute coverage across the gene and across the genome. We plotted per-base coverage divided by the total coverage across ATP2B4 exons, summed over samples within each dataset and developmental stage (Fig. 5). To compute eQTL results in *ATP2B4* exons in the 24 samples from Lessard et al.^32^, we computed fragments per kilobase of transcript per million mapped reads (FPKM) in each exon as

8 $${\mathrm{FPKM}} = \frac{{\# {\mathrm{reads}}\,{\mathrm{mapping}}\,{\mathrm{to}}\,{\mathrm{exon}} \times 10^6}}{{{\mathrm{exon}}\,{\mathrm{length}}\,{\mathrm{in}}\,{\mathrm{kb}} \times {\mathrm{total}}\,{\mathrm{number}}\,{\mathrm{of}}\,{\mathrm{reads}}}}$$

We then used a linear model to compute residual FPKM after regressing out the developmental stage (fetal or adult)^32^. For comparison across exons, we additionally standardized this residual FPKM (i.e., by subtracting the mean and dividing by the SE across samples). We computed eQTL results by fitting a linear model of genotype on the standardized, residual FPKM values.

### Analysis of ENCODE/Roadmap RNA-seq profiles

We downloaded normalized RNA-seq coverage profiles for 56 cell types, representing 12 tissues and the ENCODE cell lines, from the roadmap epigenomics browser^29^ at http://egg2.wustl.edu. We used the stranded normalized coverage files and summed across the two strand for visualization purposes (Fig. 5). To summarize coverage across tissues, we used the tissue assignments in the ‘jul2013.roadmapData.qc - Consolidated_EpigenomeIDs_summary_Table.csv’ file and computed the maximum per-base normalized coverage across all cells within each tissue across the *ATP2B4* region. ENCODE cell lines were treated as a single group, except that we treated the K562 cell line separately as it has an expression programme similar to erythroid cells.

### Generation and analysis of replication data

We selected SNPs for genotyping on the Sequenom® iPLEX Mass-Array platform (Agena Biosciences, Hamburg, Germany) as follows, following a similar protocol used previously^5^, based on a preliminary version of the GWAS analysis described above. In brief, we identified the 100 regions with the most evidence for association under a case/control model of association, using inthinnerator to identify lead variants within regions. In each region we considered up to five SNPs for replication, including the lead SNP and others with substantial evidence for association. We further ranked regions using BF_avg_ restricted to non-case/control models of association and selected additional SNPs making up to five in each region. We designed primer sets for these variants omitting those in regions previously assayed^5,7^. Altogether, we designed two multiplexes targeting 76 variants. We used these multiplexes to genotype all samples collected in the project including discovery samples and additional samples in each population, which we used for replication. Three variants had >20% missing genotyping rate across samples and were excluded from downstream analysis, leaving data for 73 variants in 35 genomic regions. Genotype calls for these assays, along with data for the SNPs previously assessed^5,7^ were combined in a single database for downstream processing and we jointly processed all the available data.

In total 37,571 individuals from this study had Sequenom genotyping available, including 15,866 SM cases, 19,845 controls and samples collected as parents. For data release purposes, we curated data across these individuals together with the 773 reference panel individuals (Fig. 1a) and 797 additional HapMap samples, and previously published trio parents for which Sequenom typing was available, for a total of 40,631 individuals. Most samples have a sex assignment by genotyping of SNPs in the X-linked gene amelogenin^5^. A subset of 955 individuals were typed from multiple blood samples in our database. For these individuals, we first removed samples with discrepancies between the clinical record of gender and the amelogenin-based sex assignment, and samples with missingness > 10% across all SNPs for which typing had been conducted. We then merged genotypes across the remaining samples for each individual to produce a single set of genotypes per individual, by taking a consensus call across the repeat typing (i.e., treating discordant calls as missing).

To provide a list of reliably typed individuals and variants, we excluded each individual with >10% missingness (across included variants for which the individual was typed) or with fewer than 50 called genotypes. Using the remaining individuals, we computed per-variant missing data rates and labelled variants with >10% missing data or fewer than 100 called genotypes for exclusion. We then iterated this procedure, updating the list of included samples and variants for two additional iterations, at which point no change in included samples or variants was observed. This process produced a total of 37,732 individuals typed across 582 variants with low rates of missing data, including 15,024 SM cases and 18,556 controls.

We separated the curated individuals into discovery and replication cohorts as follows. All individuals included in the GWAS phased genotype dataset were marked as discovery individuals (a total of 17,267 individuals). The remaining 15,548 cases and controls who were not included in discovery data and who had an assigned ethnic group as described in ‘Replication analysis’ below, were used for replication analysis.

### Validation of imputation results

For each imputed lead variant, we used QCTOOL to compute the correlation between imputed genotypes and Sequenom-typed genotypes, for all variants within 2 Mb of the lead variant, in discovery samples within each population and across samples. We treated signals as potentially replicable if a SNP with *r*^2^ > 0.75 with the lead imputed SNP across discovery samples and typed at least 1000 replication samples were present (Supplementary Data 1). We refer to such variants as Sequenom tags. A total of 25 regions among those with BF_avg_ > 1000 had a Sequenom tag. For some lead variants, multiple Sequenom tags were available. Association test power depends linearly on sample size and on the square of the correlation coefficient^95^. To assign a single ‘best tag’ for our primary replication analysis in each region, we ranked variants by the product of *r*^2^ in discovery samples and the number of non-missing genotypes in replication samples, and chose the highest-ranking SNP. Evidence at tag SNPs are presented in Supplementary Data 1 and for the SNPs with the highest levels of discovery evidence in Supplementary Figs. 5 and 6.

### Replication analysis

We used SNPTEST to compute association test results using the Sequenom genotypes, separately in discovery samples, replication samples and across all analysis samples in each population. For replication analysis, we used reported ethnic group as a covariate to control for potential confounding due to structure in each population. Specifically, we removed samples with unreported ethnic group and we grouped ethnic groups with fewer than 20 individuals into a single category named ‘OTHER’; we then included the resulting groups as a fixed effect in replication analysis. Variation in ethnic groups was present in all populations except for Malawi (where ethnicity information was not systematically collected) and Burkina Faso (where all cases and controls were collected with ethnic group ‘MOSSI’). We used BINGWA to compute a fixed-effect meta-analysis across discovery data (based on imputed genotypes, as in our discovery scan) and replication data (based on Sequenom typing) at each Sequenom-typed variant.

We extended the Bayesian analysis described above to a discovery/replication analysis. A full description of this method can be found in Supplementary Note 5, but in brief, this analysis produces a discovery BF (BF_avg_ as described above), an overall BF across discovery and replication samples (BF_overall_), and a replication BF (BF_replication_), which is computed under the posterior effect-size distribution learnt from discovery samples. These quantities satisfy

9 $${\mathrm{BF}}_{{\mathrm{overall}}} = {\mathrm{BF}}_{{\mathrm{avg}}} \times {\mathrm{BF}}_{{\mathrm{replication}}}$$

Intuitively, BF_replication_ measures the evidence for association under the set of models and effect-size distribution learnt from discovery samples. In practice, requiring replication effect sizes to match the distribution learnt from discovery may be too restrictive—e.g., this could happen if discovery effects sizes at chosen SNPs are affected by Winner’s curse or because the distribution of phenotypes in replication samples differs from that in discovery. We therefore assessed replication data assuming true effect sizes in discovery and replication samples had a correlation of 0.9. The effects of this assumption are discussed in Supplementary Note 5. To report replication results where our chosen best tag is not the lead GWAS variant, we report the BF_avg_ for the lead variant and compute BF_replication_ using the tag variant (Fig. 2c and Supplementary Data 1). We then compute BF_overall_ as the product of BF_avg_ and BF_replication_.

For rs62418762, discovery data suggests a protective effect of the reference allele on both CM and SMA susceptibility. We therefore also computed a replication *P*-value for the alternative model ‘CM and SMA effects are protective’ for this SNP. To do this, we simulated 10,000,000 parameter values under the null model, using a two-dimensional normal distribution with mean zero and covariance set to the estimated covariance matrix of the replication log-likelihood from meta-analysis. We estimated the *P*-value as the proportion of simulated parameter values, which lay in the negative quadrant and were more extreme than the observed quantities (in the sense that they are points at lower density than the observed effect sizes under this normal distribution.) This *P*-value is larger than the replication *P*-value for CM alone and is reported in Fig. 3b.

For rs62418762, we additionally investigated between-population heterogeneity in effects in discovery or replication populations by fitting a random-effect meta-analysis model. We did not observe strong evidence for heterogeneity in CM, SMA or OTHER subphenotypes either in discovery or replication data (likelihood ratio test *P* > 0.05 against null model of no between-population heterogeneity), but we note that power to detect heterogeneity is limited due to the relatively small subphenotype sample counts in some populations (Supplementary Fig. 5).

### Joint modelling of effects at associated loci

We reestimated the effect of the five replicating variants on CM, SMA and nonspecific cases, as well as on samples having both CM and SMA phenotypes, by fitting a joint model of effects across variants and populations. In addition, two other variants (rs33930165, encoding haemoglobin C (HbC), and rs8176746, which is indicative of A/B blood type, were included as they are proximal to rs334 and rs8176719, respectively, and have been previously implicated in malaria susceptibility^5,53^. To provide the most accurate estimates, in this analysis we included only the ‘strict’ set of cases having a clinical data record showing positive measured parasitaemia. We fit the model using the nnet package in R and including five principal components from each population and the study population indicator as covariates. We treated effects as additive, except that variants with a clearly non-additive effect (as in Fig. 2c) were encoded using the predicted protective dosage. Where ambiguous, we used the estimated effect on CM to determine the protective allele and for this analysis we assumed effects were fixed across populations. Results are shown in Fig. 4a. This analysis suggested little evidence of association with rs8176746, indicating that observed effects at this SNP are likely due to linkage disequilibrium with the O blood group mutation rs8176719.

We used control samples to compute the frequency of the protective dosage (or the allele frequency for variants with additive effects) for each variant across populations. To visualize frequencies, we computed the minimum, maximum and mean frequency for each variant across populations, and plotted frequencies as circles with width proportional to the maximum frequency (Fig. 4b).

To investigate potential interactions between variants, we computed the combined multilocus genotype for each sample across these variants, omitting rs8176746. Combined genotypes with <25 samples were collapsed into a single, ‘other genotype’ category (not shown). We refit the model for SM across populations using these multilocus genotypes as predictors and plotted the estimated effect sizes and confidence intervals against the expectation given the estimates from individual variants and the assumption that variants contribute independently to the log odds of disease. These estimates are shown in Fig. 4c.

### Mendelian randomization with blood cell traits

We conducted MR analysis (which, under restrictive assumptions, can provide evidence of a causal link between two traits^96^) using summary statistics for 36 RBCs, white blood cells (WBCs) and platelet traits previously published^36^. We treated each RBC, WBC or platelet trait separately. For each trait T, we assumed a model of an effect of T on SM, such that a unit increase in T increases the log odds of SM by a quantity *ρ*. If this is the case then for any genetic variant affecting T we should have

10 $$\beta _{{\mathrm{SM}}} = \rho \times \beta _{\mathrm{T}} + \zeta$$

where *β*_T_ and *β*_SM_ denote the effect of the variant on T and on SM, and ζ represents any additional effect of the variant on SM, over and above the effect through T. Under the assumptions traditionally underlying MR analysis^96^, *ζ* = 0 and we assumed this throughout.

We used a Gaussian likelihood formulation of MR^97^ under which the effect-size estimates (*β'*_T_ and *β'*_SM_) are assumed distributed around the true effects according to the estimated SEs, as:

11 $$\left( {\begin{array}{*{20}{c}} {\dot \beta _T} \\ {\dot \beta _{SM}} \end{array}} \right)\sim {\mathrm{MVN}}\left( {\begin{array}{*{20}{c}} {\beta _T} \\ {\beta _{SM}} \end{array};\left( {\begin{array}{*{20}{c}} {se_T^2} & 0 \\ 0 & {se_{SM}^2} \end{array}} \right)} \right)$$

and true effects are assumed to follow:

12 $$\left( {\begin{array}{*{20}{c}} {\beta _T} \\ {\beta _{SM}} \end{array}} \right)\sim {\mathrm{MVN}}\left( {0;\left( {\begin{array}{*{20}{c}} {\sigma _T^2} & {\rho \sigma _T^2} \\ {\rho \sigma _T^2} & {\rho ^2\sigma _T^2} \end{array}} \right)} \right)$$

The latter equation reflects Eq. (10) when *ζ* *=* *0* and the assumption that effects follow a Gaussian distribution.

To estimate *ρ*, we focused on the 2706 ‘sentinel’ variants identified as associated with blood traits by Astle et al.^36^. A subset of 2130 of these variants were included in the meta-analysis in our study after filtering by EMAC. We removed 89 variants lying in any of the 95 regions of association containing a variant with BF_avg_ > 1 × 10^3^ in our discovery analysis (including the *ATP2B4* region). We treated the remaining variants as independent and estimated *ρ* and *σ*^2^_T_ by minimizing Eq. (12) using the optim() function in R. To compute a *P*-value for model fit, we refit the model assuming no correlation (*ρ* = 0) and computed the likelihood ratio *P*-value.

We visualized effect size and the model fit by plotting the effect-size estimates on T and on SM across variants, overlaid with a line indicating the estimated value of *ρ* and its 95% confidence interval. To avoid visualization problems stemming from large estimates at variants with high SEs, we used effect-size estimates regularized using a N(0, 0.1) prior in plots. These estimates are plotted in Fig. 5h (for mean corpuscular haemoglobin) and Supplementary Fig. 12 (for all traits tested).

For T *=* MCHC, our estimate of effect variance was *σ*^2^_T_ = 0.013^2^. We plotted the empirical distribution of observed effects overlaid with the Gaussian density and observed some evidence that effects are overdispersed relative to the Gaussian distribution with this variance. To verify that this did not adversely affect results, we refit the model assuming a fixed prior value *σ*^2^_T_ = 0.1^2^, with similar results (*ρ* = − 0.13, *P* = 7 × 10^−3^).

### HLA region analysis

We tested for association and conducted meta-analysis for imputed HLA alleles in the same way as described above for other variants. To compare imputed HLA allele calls with previously published antigen frequencies, we first computed a predicted antigen frequency for each allele as the proportion of individuals heterozygote or homozygote for the allele. We plotted these frequencies in control samples from each Gambian ethnic group against previously published HLA Class I antigen frequencies (Table 1 of Hill et al.^51^). One pair of alleles were particularly discordant (the B*15 allele, which is at relatively high frequency in our imputation and the B70 antigen, which is at relatively high frequency in the serotyped data). We used the IPD-IMGT/HLA dictionary to confirm that the B70 antigen is expressed by B*15 alleles and we therefore computed the combined B70/B15 frequencies in Fig. 6b. For each ethnic group, we also computed squared correlation between imputed and serotyped antigen frequencies.

To further assess HLA imputation, we selected 31 Gambian case samples, for which both parents have previously been genotyped (EGA dataset EGAD00000000019) for HLA typing based on available DNA quantities. These samples were typed at 11 HLA gene loci through exon sequencing. Sequenced regions included exons 2–4 of HLA-A, -B, -C, -DPB1, exons 2–3 of HLA-DPA1, -DQA1, DQB1 and DRB1, and exon 2 of HLA-DRB3, -DRB4 and -DRB5. Typing was performed by the Accredited Tissue Typing Laboratory at Addenbrooke’s Hospital, Cambridge University Hospitals NHS Foundation Trust, using the proprietary uTYPE software version 7 (Fisher Scientific, Pittsburgh, USA). The list of possible ambiguous calls were minimized by using the ‘allele pair’ export function in this software, which lists all possible and permissible allele pair possibilities for each locus for each individual. Alleles were defined using the IMGT/HLA Release: 3.22.0 2015 October 10. Best-call allele pairs for each locus in each individual were determined based on local guidelines prioritizing alleles that were denoted Common and Well-Documented.

### Analysis of glycophorin structural variants

We tested for association and conducted meta-analysis for glycophorin region variants in the same way as described above for other variants. Four CNVs were imputed with reasonable frequency and imputation quality (DUP1; the glycophorin B deletions DEL1 and DEL2, and DUP4). We note that glycophorin region SNPs appear in our meta-analysis results twice—once using the genome-wide combined reference panel and once based on this imputation of glycophorin variants. In our main presentation for consistency, we refer to the genome-wide panel imputation results, except for glycophorin CNVs, which are imputed from the glycophorin panel.

### Analysis of polygenicity of severe malaria

We aimed to assess the degree to which additional polygenic effects explain SM susceptibility. The epidemiology of malaria differs between sub-Saharan Africa and elsewhere, and we chose to restrict attention to the African populations in our data for this analysis. We further restricted attention to a subset of 13,088 samples having pairwise relatedness < 0.05 (estimated in each population based on the kinship matrix used to compute PCs from phased genotypes, as described above). We used the genotypes at directly typed SNPs in the phased dataset for heritability estimation.

We used two previously published methods (GCTA^98^ and PCGC^22^, in the implementation by Gaurav Bhatia (https://github.com/gauravbhatia1/PCGCRegression)), to estimate the heritability of SM. These methods are both based on the infinitesimal model of genotype-trait association, under which all variants contribute equally, in expectation, to trait variation. However, they differ in how estimation is performed. In particular, PCGC is thought to be more robust for case/control traits and we use it for our main results.

We estimated heritability based on the phased dataset in several ways:

- in each chromosome separately
- in a joint model in which all chromosome were included as separate components
- in a joint model in which the four previously confirmed association regions and the rest of the genome were included as separate components
- in a joint model in which regions near protein-coding genes and the rest of the genome were included as separate components
- in a joint model in which variants in different minor allele frequency bins were allocated to different components.

For our main analysis, we included an indicator of country and 20 PCs, computed across the 13,088 samples, as covariates. GCTA takes covariates into account directly; to adjust for covariates in PCGC, we used the --adjust-grm option in LDAK^25^ to subtract covariates from the relatedness matrix (however, we did not further explore using the LDAK model for heritability estimation). For some analyses, we also included the protective dosages of risk alleles at HbS, rs8176719 (*ABO*), rs4951377 (*ATP2B4*) and DUP4 as covariates. We also explored including 10 or 50 PCs as covariates, with little difference in results. We observed slightly smaller estimates of heritability using GCTA than PCGC, consistent with the previously reported theory and observations^22^. Although our populations are highly structured, estimated of per-chromosome heritability when fitting chromosomes independently or jointly were similar (Supplementary Fig. 4). This suggests that any between-chromosome correlations between variants, which can occur as a result of population structure, are being adequately controlled for by our inclusion of covariates.

### Between-continent allele frequency differentiation

For a malaria-associated variant, the malaria hypothesis suggests that natural selection will maintain the protective allele at higher frequency in populations of high childhood mortality (such as African populations in our study) due to malaria. To assess this while avoiding confounding by selection of variants through our GWAS (conducted in a sample that is largely from sub-Saharan Africa), we aimed to condition on the African allele frequency and ask whether the allele is at lower frequency in non-endemic populations. We first computed the empirical allele count distribution between the African continental group (AFR) and non-African continental groups G (where G = EUR or EAS) in the reference panel, based on all variants which had an ancestral allele assignment and which were not masked by the 1000 Genomes strict mask. Let **D** = (*d*_*ij*_) be the matrix of derived allele counts, where i ranges from 0 to the number of haplotypes in G and j ranges from 0 to the number of haplotypes in AFR. For any given derived allele *x*, we computed upper and lower ranks of the allele in G, conditional on its count in AFR, as

13 $${\mathrm{rank}}_{\mathrm{G}}^ + \left( x \right) = \left( {\mathop {\sum}\limits_{{\mathrm{i}} > {\mathrm{a}}} {d_{{\mathrm{ib}}} + 1/2d_{{\mathrm{ab}}}} } \right)/\left( {\mathop {\sum}\limits_i {d_{{\mathrm{ib}}}} } \right)$$

and

14 $${\mathrm{rank}}_{\mathrm{G}}^ - \left( x \right) = \left( {\mathop {\sum}\limits_{{\mathrm{i}} < {\mathrm{a}}} {d_{{\mathrm{ib}}} + 1/2d_{{\mathrm{ab}}}} } \right)/\left( {\mathop {\sum}\limits_i {d_{{\mathrm{ib}}}} } \right)$$

where *a* is the number of *x* alleles in G and b is the number of *x* alleles in AFR. These quantities can thus be interpreted as the upper and lower rank of the allele *x* among all derived alleles with the same allele count in AFR, and satisfy rank^+^_G_(*x*) + rank^−^_G_(*x*) = 1. To compute the African allele counts in *D*, we used only samples from non-admixed African populations (i.e., excluding ACB and ASW populations).

For any variant *v* associated with malaria, let *x* be the derived allele of *v*. We defined rank_G_(*v*) as rank^−^_G_(*x*) if *x* is associated with decreased risk, or rank^+^_G_(*x*) if *x* is associated with increased risk. Where unclear, we determine the direction of effect of *x* based on the effect size estimated for CM. Fig. 7a depicts rank_EUR_(*v*) for the 91 autosomal variants with BF_avg_ > 1000, which have an ancestral allele assignment in the 1000 Genomes ancestral allele sequence. We also annotated variants genome-wide with rank_EUR_ and rank_EAS_.

### Within-Africa allele frequency differentiation

To assess differentiation within Africa, we implemented a model similar to Bayenv^99^ but with simplifications that we now describe. The Bayenv model assumes that the vector of underlying allele frequencies in sampled populations (denoted **F**) follows a multivariate normal distribution of the form

15 $${\mathbf{F}}\sim {\mathrm{MVN}}(f_0,f_0(1 - f_0){\mathbf{\Omega }})$$

Here, *f*_0_ denotes the conceptual ancestral allele frequency (assumed to reflect a time point *T* before the populations from which study samples were drawn separated) and the matrix **Ω** captures the effects on allele frequency of the co-ancestry of populations since *T*. In particular, diagonal entries of **Ω** reflect levels of genetic drift (relative to the population at time point *T*) and off-diagonal entries reflect shared ancestry between populations.

The model above is expected to hold approximately provided *f*_0_ is not too small and the levels of genetic drift are not too large^99^. As the co-ancestry matrix **Ω** is assumed to be the same across all neutrally evolving variants, it can be estimated from a set of neutral SNPs across the genome. Specifically, for each variant, a vector of scaled frequencies can be computed as

16 $${\mathbf{F}}^\prime = ({\mathbf{F}} - {\mathrm{ }}f_0)/\surd (f_0(1 - f_0))$$

Under the model above, Ω can now be estimated as the covariance of scaled allele frequencies, **Ω** *=* cov(**F′**).

To implement this in practice, we made the following simplifying assumptions. First, we restricted attention to the 7 African study populations with >200 control samples (namely Gambia, Burkina Faso, Ghana, Cameroon, Malawi, Tanzania and Kenya) and assumed that the population allele frequency vector **F** is accurately estimated by the observed allele frequencies (using only control samples). Second, we assumed that the ancestral allele frequency *f*_0_ is well estimated as the mean of the per-population frequency estimates. Third, we restricted attention to directly typed SNPs with mean allele frequency in the range 2–98% and we assumed that the effects of allele frequencies reaching frequency 0 or 1, which is not explicitly modelled in the above framework, would be minor. We sampled a subset of 100,000 such SNPs randomly across the genome with the above properties and computed **Ω** as the empirical covariance in scaled estimated allele frequencies. The estimated matrix **Ω'** is visualized in Supplementary Fig. 9. We note that the Bayenv method avoids these assumptions by integrating over the uncertainty in ancestral and current allele frequencies^99^, and by modelling the population frequencies as bounded between 0 and 1, but requires a computationally expensive Markov Chain Monte-Carlo (MCMC) process.

In practice, systematic effects in **F'** may arise due to the selection of variants used in estimation. In addition to **Ω'**, we therefore additionally estimated the mean scaled frequency, denoted *μ*.

The estimated covariance matrix **Ω'** provides a model for allele frequency variation of putatively neutral alleles in the populations considered. We visualized this model by plotting the joint distribution of observed and simulated allele frequencies. Specifically, we simulated 1,000,000 variants by sampling *f*_0_ from the observed list of mean allele frequencies and then sampling allele frequencies from Eq. (15). We then plotted the joint allele frequency distribution in the two most divergent populations, Gambia and Kenya (Supplementary Fig. 9).

For each typed or imputed variant, we tested for differentiation against this model as follows. We first computed the mean allele frequency and scaled allele frequency vector **F'** for the variant. The model above states that **F′** ~ MVN(*μ*, **Ω**'), or equivalently

17 $${\mathbf{X}}\sim {\mathrm{MVN}}(0,{\mathbf{Id}})$$

where **X** *=* **L**^*−1*^ *(***F′** − *μ)* and **LL**^t^ ^*=*^ **Ω′** denotes the Cholesky decomposition of the estimated matrix **Ω'**. In practice, the use of mean allele frequency implies that **Ω'** has rank one less than the number of populations^99^ and we therefore remove one population from the vector F' and from **Ω**' before computing this decomposition. In the results presented here, we chose to remove Cameroon, which is the most central population both geographically and within **Ω** (Supplementary Fig. 9). Under the model, the sum of squared entries of **X**, i.e., **X**^t^**X**, is now distributed as *χ*^2^-distribution with 6 degrees of freedom. We denote the corresponding *P*-value as P_XtX_.

Supplementary Fig. 10 compares the distribution of the **X**^**t**^**X** test statistic against the *χ*^2^-distribution for typed and imputed variants at different frequencies. Supplementary Fig. 11 depicts -log10 *P*_XtX_ for all imputed variants with mean allele frequency in the range 2–98%.

### Reporting summary

Further information on research design is available in the Nature Research Reporting Summary linked to this article.

## Supplementary information

Description of Additional Supplementary Files

Supplementary Information

Supplementary Data 1

Supplementary Data 2

Supplementary Data 3

Supplementary Data 4

Supplementary Data 5

Reporting Summary

## Data Availability

Illumina Omni 2.5 M genotype data from study samples (Fig. 1a, right panel), corresponding phased and imputed datasets, genotypes generated on the Sequenom iPLEX Mass-Array platform for selected variants in discovery and replication samples, and HLA allele genotypes for 31 Gambian individuals (Supplementary Data 5) have been deposited in the European Genome-Phenome Archive (EGA; study accession EGAS00001001311). Whole-genome sequence read data for samples from Burkina Faso, Cameroon and Tanzania (Fig. 1a, left panel) have been deposited in the EGA (study accession EGAS00001003648). Access to MalariaGEN datasets on EGA is by application to an independent data access committee. Sequence read data for Gambian Genome Variation Project samples (Fig. 1a, left panel) is available under open access terms through the European Nucleotide Archive (PRJEB3013 (Fula), PRJEB3252 (Jola), PRJEB1682 (Mandinka) and PRJEB1323 (Wollof)) and the 1000 Genomes Project data portal (https://www.internationalgenome.org). A full set of association summary statistics underlying our analysis are available for download through the MalariaGEN website (https://www.malariagen.net/resource/25) and the NHGRI-EBI GWAS Catalog (https://www.ebi.ac.uk/gwas/downloads/summary-statistics). The source data underlying Figs. 2a, 3, 4, 7 and Supplementary Figs. 5–7 are provided as a Source Data file. Further details of data and additional resources underlying this manuscript can be found on the MalariaGEN website (https://www.malariagen.net/resource/25).

## Source Data

Source Data

## Code availability

Software underlying the analyses in this paper and further resources are available at http://www.malariagen.net/resource/25.
