# Supplementary material for: Insights into malaria susceptibility using genome-wide data on 17,000 individuals from Africa, Asia and Oceania
Source: Nat Commun. 2019 Dec 16;10:5732. doi: 10.1038/s41467-019-13480-z (PMC6914791; doi:10.1038/s41467-019-13480-z)
Supplement: Supplementary file 1 — Description of Additional Supplementary Files [file 41467_2019_13480_MOESM1_ESM.pdf]

Description of Additional Supplementary Data files

Supplementary Data 1 - The 97 regions with BF_{avg} > 1,000

The table reflects the 97 regions containing a variant with BF_{avg} > 1000 in our data, identified chosen by using the inthinnerator tool applied to variants ranked by BF_{avg}, as described in **Methods**. Columns represent: chromosome, position, rsid, reference and non-reference alleles of the lead variant; details of the association region including the region boundaries, nearest gene, distance to the nearest gene, and all genes in the association region; detail of our Bayesian meta-analysis, including Bayes factors (BFs) for case/control and subphenotype models of association (each BF is averaged over the component models detailed in **Methods** and **Supplementary Table 5**) for four modes of inheritance, the BF_{avg} and best posterior model, detail of population- or group-specific BFs included in the case-control BF (each BF is averaged over four modes of inheritance); detail of fixed-effect meta-analysis including effect size estimates, standard errors, and Wald test p-values for each parameter under the case-control and multinomial models, for each mode of inheritance. Columns ending 'included' contain strings indicate which per-population effect size estimates are included in the meta-analysis, where 1 indicates included and 0 indicates excluded due to criteria outlined in Methods, with populations in the order depicted in **Figure 1a**; for subphenotype meta-analysis there are 3 characters per population indicating the three estimated parameters. Subsequent columns provide details of replication analysis, where applicable, including the number of Sequenom tags, detail of the best tag, correlation between imputed and directly-typed genotypes for the best tag measured across discovery samples, the total count of non-missing genotype calls for the best tag in replication samples, the overall and replication BF, two-sided replication P-value under the case-control and subphenotype models for each mode of inheritance, and details of other available Sequenom tags where applicable.

Supplementary Data 2 - Variants showing heterogeneous patterns of association

A list of variants having heterogenous patterns of estimated effects across populations, identified as having maximum BF (BF_{max}) > 25,000 and at least 100 times greater than the BF under a fixed-effect model. Only an additive model test of association with case/control outcome is considered. The maximum is computed across all models tested, which include those in **Supplementary Table 5** and additional population and group-specific models, including population-specific effects. We restrict to variants with an effective minor allele count of at least 1,706 (corresponding to a minor allele frequency of 5% across all study samples for well-imputed SNPs). We removed variants in the *HBB* and glycophorin regions. Columns represent the identifier, chromosome, position, rsid, and alleles of the variant, and an indicator of whether the variant was imputed from the combined panel ('gwas') or the 1000 Genomes panel ('1000GP'); an indicator of which populations contributed to the meta-analysis, the total meta-analysis sample size, and the effective minor allele count; the BF_{avg} and best and second best posterior model; the maximum BF across all models tested and the model showing the maximum BF; all component BFs (model names include a string of 0's and 1's indicating assumed zero or nonzero effects in each population as described in **Methods**); and between-continent and within-Africa differentiation metrics.

Supplementary Data 3 - Heritability estimates

The table shows heritability estimates made using PCGC [22] and GCTA [23] based on directly-typed genotypes in our QCd set of data. Estimates are made based on 13,030 samples from African study populations chosen to have relatedness < 0.05 within populations. We computed principal components across this set of samples and include an indicator of study site and 10, 20, or 50 PCs as covariates to allow for potential confounding by major axes of population structure. We present results for estimates across the whole genome, joint estimates across all chromosomes, estimates of contributions from chromosomes estimated independently, estimates split into regions of replicable associations and the remainder of the genome, and split by variant frequency, as denoted by the first column. Additional columns indicate the covariates included, the subset of SNPs for which the estimate applies (where 'combined' denotes a sum over all other components in the analysis), the number of SNPs included in the subset, the estimated liability scale heritability from PCGC and GCTA and the corresponding

standard errors, and the estimated proportion of heritability per SNP. A subset of these estimates is visualized in **Supplementary Figure 4**.

Supplementary Data 4 - Functional annotation for variants in 95% credible set of top 97 association regions

The table reflects identified functional annotations of variants in the 95% credible set of the regions in **Supplementary Table 2**, where the credible set is computed assuming a single causal variant is present (i.e. by reweighting BF_{avg} across variants). Variants with BF_{avg} < 100 are excluded from the table. Columns reflect the id, rsid and BF_{avg} of the lead variant in the region; the id, chromosome, position, rsid, BF_{avg}, and best posterior model at the annotated variant; indicators of whether the variant lies in a protein coding gene and/or in an exon of a protein coding gene; the gene name where applicable; the output of Variant Effect Predictor; ENCODE transcription factor binding sites the variant lies in; an indicator of whether the variant lies in a GATA1 or TAL1 motif; inferred chromatin states at the variant in selected cell types, from Roadmap Epigenomics Project data; the mean allele frequency and P-value for the XtX test of population differentiation; the rank of the count of the estimated protective allele in European ($rank_{EUR}$) and east Asian ($rank_{EAS}$) reference panel populations, conditional on the observed count across African populations; genes for which the variant has been identified as an eQTL in peripheral blood [24], GTEx tissues [25], or erythrocyte precursors [26]; RBC trait associations [27]; and GWAS trait associations [28].

Supplementary Data 5 - Summary of HLA typing

The table presents a comparison of HLA classical allele genotypes determined by HLA typing and by imputation, in 31 Gambian children who are cases in our study. Columns represent the HLA locus and allele; counts of samples typed with each genotype for the allele (which we consider to be true genotypes); counts of samples imputed with each genotype for the allele (using a cutoff of 0.75 probability where imputation is uncertain); counts of samples of each true genotype wrongly imputed; counts of samples of each imputed genotype wrongly imputed; and the correlation, recall and precision of the imputed genotypes.
