# Supplementary material for: Insights into malaria susceptibility using genome-wide data on 17,000 individuals from Africa, Asia and Oceania
Source: Nat Commun. 2019 Dec 16;10:5732. doi: 10.1038/s41467-019-13480-z (PMC6914791; doi:10.1038/s41467-019-13480-z)
Supplement: Supplementary file 8 — Reporting Summary [file 41467_2019_13480_MOESM8_ESM.pdf]

nature research

Corresponding author(s): Gavin Band

Last updated by author(s): 22/10/19

Reporting Summary

Nature Research wishes to improve the reproducibility of the work that we publish. This form provides structure for consistency and transparency in reporting. For further information on Nature Research policies, see Authors & Referees and the Editorial Policy Checklist.

Statistics

For all statistical analyses, confirm that the following items are present in the figure legend, table legend, main text, or Methods section.

n/a	Confirmed	
☐	☒	The exact sample size (*n*) for each experimental group/condition, given as a discrete number and unit of measurement
☐	☒	A statement on whether measurements were taken from distinct samples or whether the same sample was measured repeatedly
☐	☒	The statistical test(s) used AND whether they are one- or two-sided<br>*Only common tests should be described solely by name; describe more complex techniques in the Methods section.*
☐	☒	A description of all covariates tested
☐	☒	A description of any assumptions or corrections, such as tests of normality and adjustment for multiple comparisons
☐	☒	A full description of the statistical parameters including central tendency (e.g. means) or other basic estimates (e.g. regression coefficient) AND variation (e.g. standard deviation) or associated estimates of uncertainty (e.g. confidence intervals)
☐	☒	For null hypothesis testing, the test statistic (e.g. *F*, *t*, *r*) with confidence intervals, effect sizes, degrees of freedom and *P* value noted<br>*Give P values as exact values whenever suitable.*
☐	☒	For Bayesian analysis, information on the choice of priors and Markov chain Monte Carlo settings
☐	☒	For hierarchical and complex designs, identification of the appropriate level for tests and full reporting of outcomes
☐	☒	Estimates of effect sizes (e.g. Cohen's *d*, Pearson's *r*), indicating how they were calculated

Our web collection on statistics for biologists contains articles on many of the points above.

Software and code

Policy information about availability of computer code

Data collection	No software was used for data collection.

Data analysis

For data analysis and presentation in this study we developed the following software:

- QCTOOL v2.0 (http://www.well.ox.ac.uk/~gav/qctool)
- SNPTEST v2.5.4 (http://www.well.ox.ac.uk/~gav/snptest)
- BINGWA v1.0 (http://www.well.ox.ac.uk/~gav/bingwa)
- INTHINNERATOR v2.0.5 (http://www.well.ox.ac.uk/~gav/inthinnerator)

The above software is available through respective web pages. All except SNPTEST is open source.

In addition we used a number of software packages by other authors, including:

- R with packages as detailed in Methods.
- Python version 2.7 and 3.5
- sqlite versions 3.11 - 3.24
- GCTA 1.24.4 (https://cnsgenomics.com/software/gcta/)
- PCGCRegression.jar (https://github.com/gauravbhatia1/PCGCRegression)

And for analysis of sequenced data:
- GATK 3.3.0 (https://software.broadinstitute.org/gatk/)
- BEAGLE v4.0 (https://faculty.washington.edu/browning/beagle/beagle.html)
- SHAPEIT2 (https://mathgen.stats.ox.ac.uk/genetics_software/shapeit/shapeit.html)

For manuscripts utilizing custom algorithms or software that are central to the research but not yet described in published literature, software must be made available to editors/reviewers. We strongly encourage code deposition in a community repository (e.g. GitHub). See the Nature Research guidelines for submitting code & software for further information.

Data

Policy information about availability of data

All manuscripts must include a data availability statement. This statement should provide the following information, where applicable:
- Accession codes, unique identifiers, or web links for publicly available datasets
- A list of figures that have associated raw data
- A description of any restrictions on data availability

Illumina Omni 2.5M genotype data from study samples (Figure 1a, right panel), and corresponding phased and imputed datasets have been deposited in the European Genome-Phenome Archive (EGA) under study accession EGAS00001001311. Whole-genome sequence read data for Gambian Genome Variation Project samples (Figure 1a, left panel) is available through the European Nucleotide Archive under PRJEB3013 (Fula), PRJEB3252 (Jola), PRJEB1682 (Mandinka), and PRJEB1323 (Wollof). Read data for sequenced samples from Burkina Faso, Cameroon, and Tanzania (Figure 1a, left panel) have been deposited in the EGA under study accession EGAS00001003648. Genotypes generated on the Sequenom iPLEX Mass-Array platform for selected variants in discovery and replication samples, and HLA allele genotypes for 31 Gambian individuals (Supplementary Data 5) will be deposited in the EGA. A full set of association summary statistics underlying our analysis are available through the MalariaGEN website (https://www.malariagen.net/resource/25) and the NHGRI-EBI GWAS Catalog (https://www.ebi.ac.uk/gwas/downloads/summary-statistics).The source data underlying Figures 2a, 3-4, 7, and Supplementary Figures 5-7 are provided as a Source Data file.

Field-specific reporting

Please select the one below that is the best fit for your research. If you are not sure, read the appropriate sections before making your selection.

☒ Life sciences ☐ Behavioural & social sciences ☐ Ecological, evolutionary & environmental sciences

For a reference copy of the document with all sections, see nature.com/documents/nr-reporting-summary-flat.pdf

Life sciences study design

All studies must disclose on these points even when the disclosure is negative.

Sample size	We aimed to maximise sample size subject to logistical constraints. For the discovery analysis, all samples having a sufficient quantity and quality of DNA sample were included.
Data exclusions	Data quality control processes were implemented to remove low-quality samples and low-quality genetic variants prior to analysis, as described in Methods.
Replication	We attempted to replicate signals identified in the discovery phase by directly typing selected genetic variants in an independent set of >15,000 samples from the same study populations.
Randomization	Samples were recruited based on their status as a disease case or control. Sample genotype was not known at the point of recruitment to the study.
Blinding	Investigators were not aware of sample genotype at the point of recruitment to the study.

Reporting for specific materials, systems and methods

We require information from authors about some types of materials, experimental systems and methods used in many studies. Here, indicate whether each material, system or method listed is relevant to your study. If you are not sure if a list item applies to your research, read the appropriate section before selecting a response.

Materials & experimental systems

n/a	Involved in the study
☒	☐ Antibodies
☒	☐ Eukaryotic cell lines
☒	☐ Palaeontology
☒	☐ Animals and other organisms
☐	☒ Human research participants
☐	☒ Clinical data

Methods

n/a	Involved in the study
☒	☐ ChIP-seq
☒	☐ Flow cytometry
☒	☐ MRI-based neuroimaging

Human research participants

Policy information about studies involving human research participants

Population characteristics	Study samples reflect local populations. Characteristics of study samples were detailed in MalariaGEN, "Reappraisal of known malaria resistance loci in a large multicenter study", Nature Genetics (2014).
Recruitment	Disease cases were recruited on entry to local hospitals. Controls were recruited either as new births (i.e. cord blood) from local hospitals, individuals with similar age distribution from local populations, or as young adults from local populations. Full details of sample recruitment by project partners are given on the MalariaGEN webpage (https://www.malariagen.net/projects/consortial-project-1#partner-studies). Given the potential severity of malarial disease, and the geographic, genetic and cultural structure of local populations, sampling bias due to variation in rates of attendance at local hospitals is possible.
Ethics oversight	Ethics approval was given by local ethics boards. Full details of ethics approval can be found in Supplementary Table 1

Note that full information on the approval of the study protocol must also be provided in the manuscript.

Clinical data

Policy information about clinical studies

All manuscripts should comply with the ICMJE guidelines for publication of clinical research and a completed CONSORT checklist must be included with all submissions.

Clinical trial registration	*Provide the trial registration number from ClinicalTrials.gov or an equivalent agency.*
Study protocol	*Note where the full trial protocol can be accessed OR if not available, explain why.*
Data collection	*Describe the settings and locales of data collection, noting the time periods of recruitment and data collection.*
Outcomes	*Describe how you pre-defined primary and secondary outcome measures and how you assessed these measures.*
